# Supplementary material for: Long-term in vitro 2D-culture of SDHB and SDHD-related human paragangliomas and pheochromocytomas
Source: PLoS One. 2022 Sep 30;17(9):e0274478. doi: 10.1371/journal.pone.0274478 (PMC9524698; doi:10.1371/journal.pone.0274478)
Supplement: S1 Fig — (PDF) [file pone.0274478.s001.pdf]

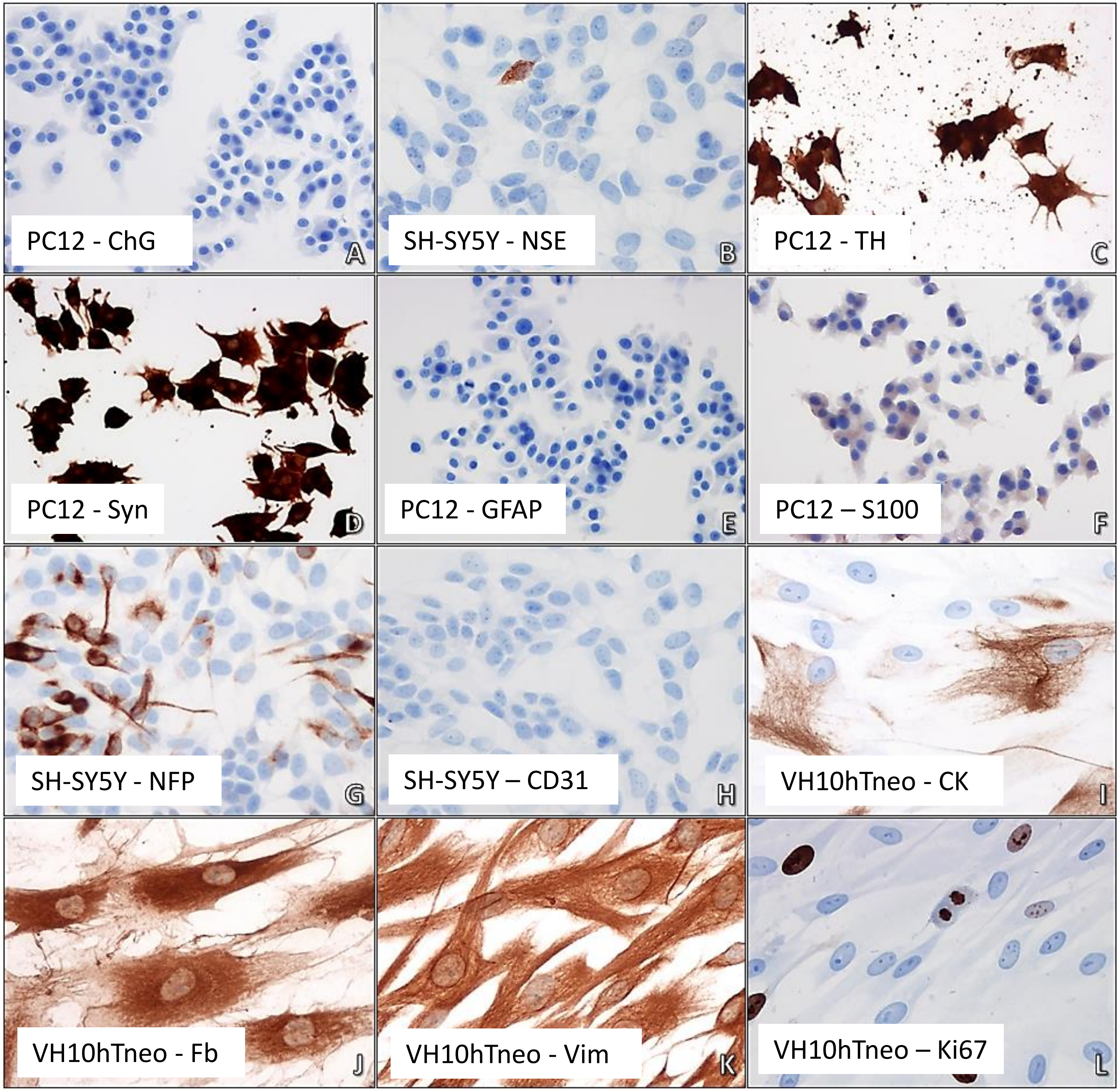
PC12 - ChG
A
SH-SY5Y - NSE
B
PC12 - TH
C
PC12 - Syn
D
PC12 - GFAP
E
PC12 – S100
F
SH-SY5Y - NFP
G
SH-SY5Y – CD31
H
VH10hTneo - CK
I
VH10hTneo - Fb
J
VH10hTneo - Vim
K
VH10hTneo – Ki67
L

S1 Fig. Overview of immunocytochemical staining for protein markers in control cell lines PC-12, SH-SY5Y and VH10hTneo. (A) α-chromogranin A (diluted 1: 1000) staining of PC-12 cells. (B) α-neuron-specific enolase (diluted 1: 2000) staining of SH-SY5Y cells. (C) α-tyrosine hydroxylase (diluted 1: 2000) on PC-12 cells (D) α-synaptophysin (diluted 1: 1000) staining of PC-12 cells. (E) α-Glial Fibrillary Acidic Protein (GFAP) (diluted 1: 1000) staining of PC-12 cells. (F) Type II sustentacular cell marker α-S100 (diluted 1: 2000) staining of PC-12 cells. (G) Neuronal cell marker α- neurofilament protein (diluted 1: 200) staining of SH-SY5Y cells. (H) Endothelial cell marker α-CD31 (diluted 1:20) staining of SH-SY5Y cells. (I) Intermediate filament marker α-cytokeratin (diluted 1: 300) staining of VH10hTneo cells. (J) Fibroblast marker α-fibronectin (diluted 1: 4000) staining of VH10hTneo cells. (K) Fibroblast marker α-vimentin (diluted 1: 1000) staining of VH10hTneo cells. (L) Proliferation marker α-Ki-67 (diluted 1: 1000) staining of VH10hTneo cells.
