# Supplementary material for: Long-term in vitro 2D-culture of SDHB and SDHD-related human paragangliomas and pheochromocytomas
Source: PLoS One. 2022 Sep 30;17(9):e0274478. doi: 10.1371/journal.pone.0274478 (PMC9524698; doi:10.1371/journal.pone.0274478)

S2.1 Fig. Summary of tumour explant culture to determine cellularity of explants. Detail [Tu9, CBT right, SDHD. H&E, 10x obj.]. Tumour explants (minced tumour fragments) were cultured for up to 39 days to assess cellularity and remaining ‘chromaffin-like’ tumour cells. Around day 11 tumour fragments begin to show clear signs of declining cellularity and an increase in eosin-avid (pink) acellular proteinaceous material. By day 39 few or no cells of chromaffin appearance remain in the tumour fragments.

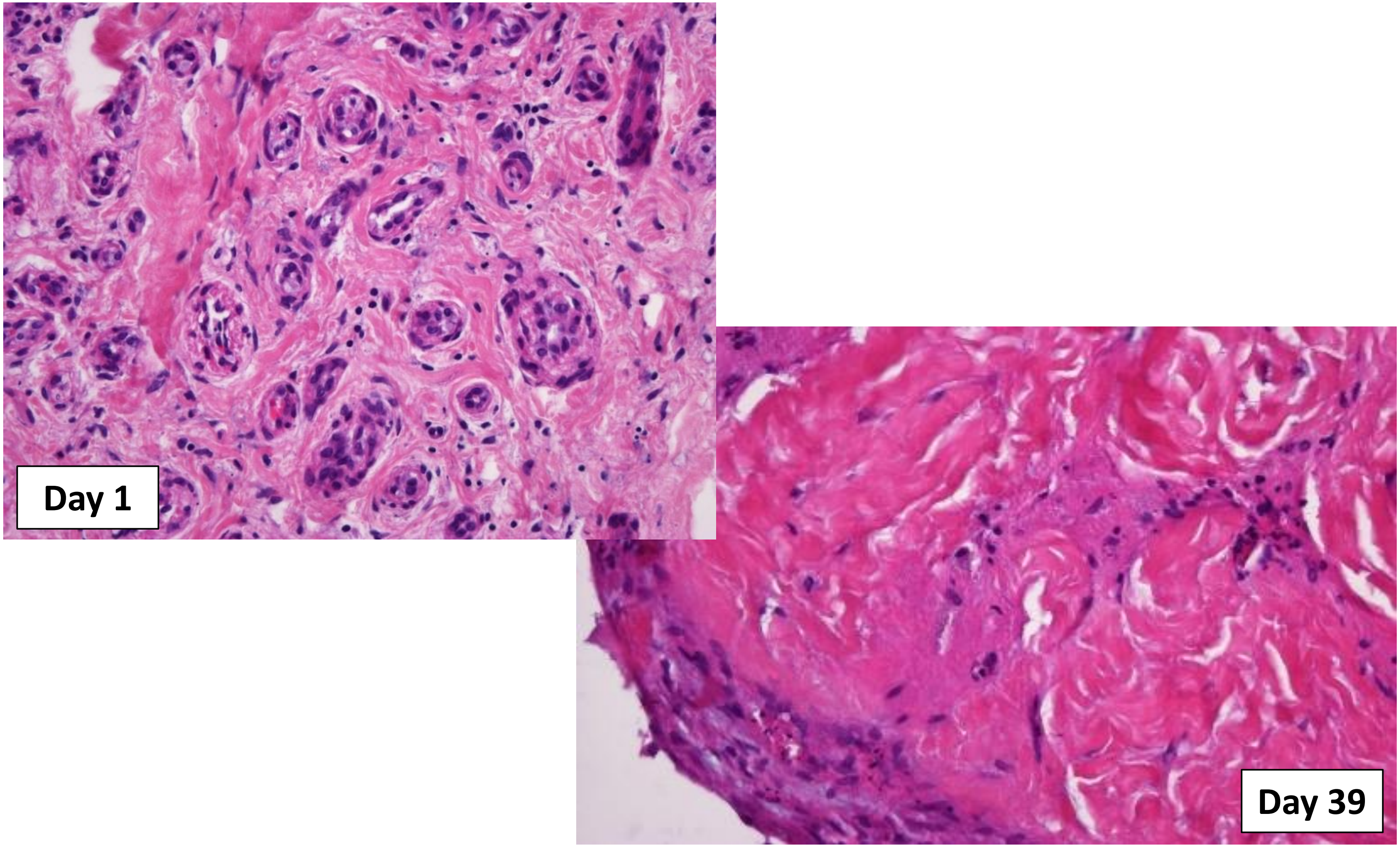

S2.2 Fig. Tumour explant culture to determine cellularity of explants. [Tu9, CBT right, SDHD. H&E, 10x obj.]. Tumour explants (minced tumour fragments) were cultured for up to 39 days to assess cellularity and remaining 'chromaffin-like' tumour cells. Around day 11 tumour fragments begin to show clear signs of declining cellularity and an increase in eosin-avid (pink) acellular proteinaceous material. By day 39 few or no cells of chromaffin appearance remain in the tumour fragments.

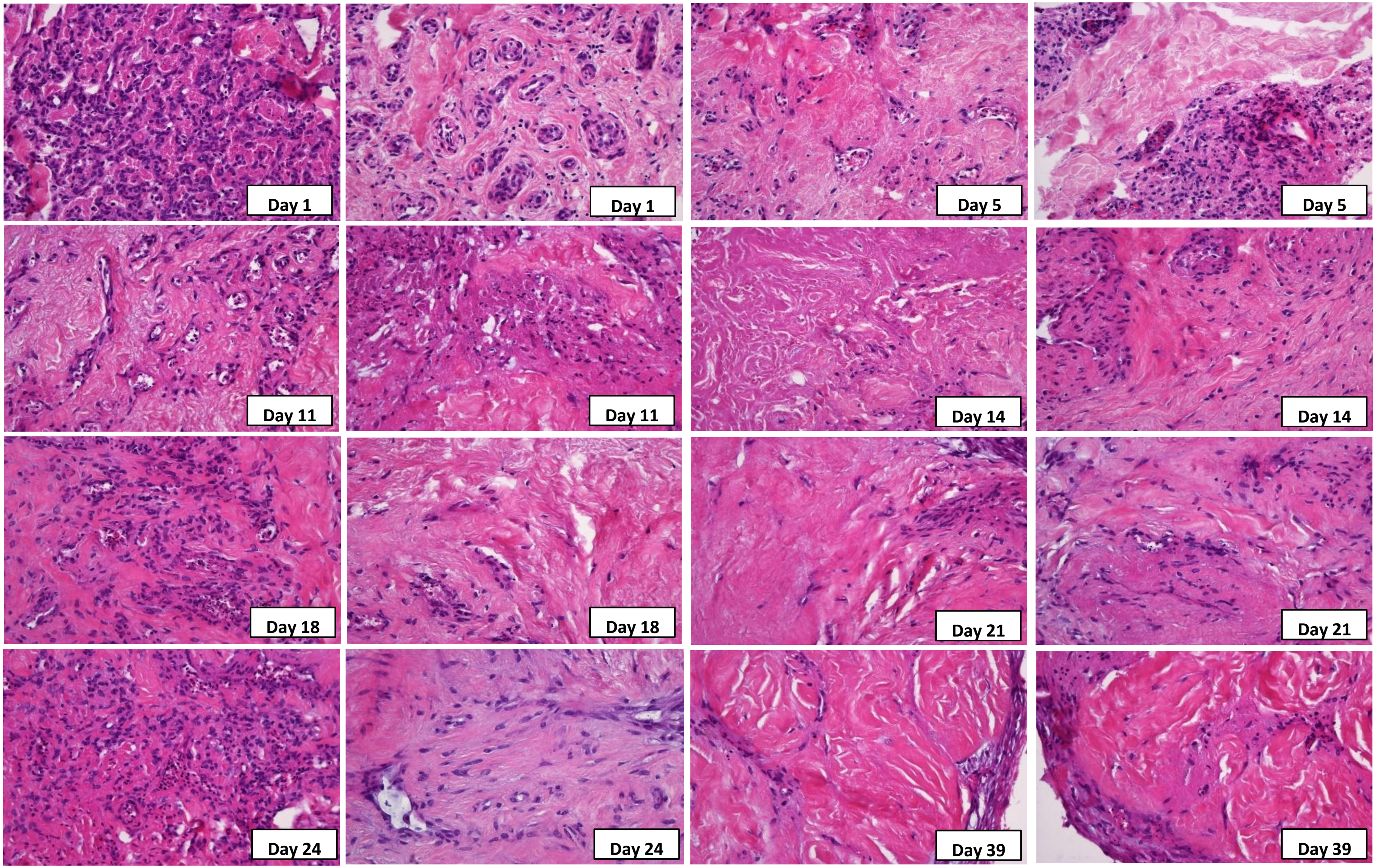

S2.3 Fig. Tumour explant culture to determine cellularity of explants . Detail [Tu44, CBT right, SDHD]:
Tumour explants (minced tumour fragments) cultured for up to 32 days to assess cellularity and proportion of synaptophysin-positive cells ('chromaffin' tumour cells) remaining. By day 32, declining expression of synaptophysin is accompanied by visible shrinkage of tumour areas. Residual chromaffin cell areas can been seen but the lack of nucleic acid (DNA) staining by haematoxylin suggests a loss of cellular integrity and that the areas lightly-staining for synaptophysin consist of cellular debris of chromaffin cells.

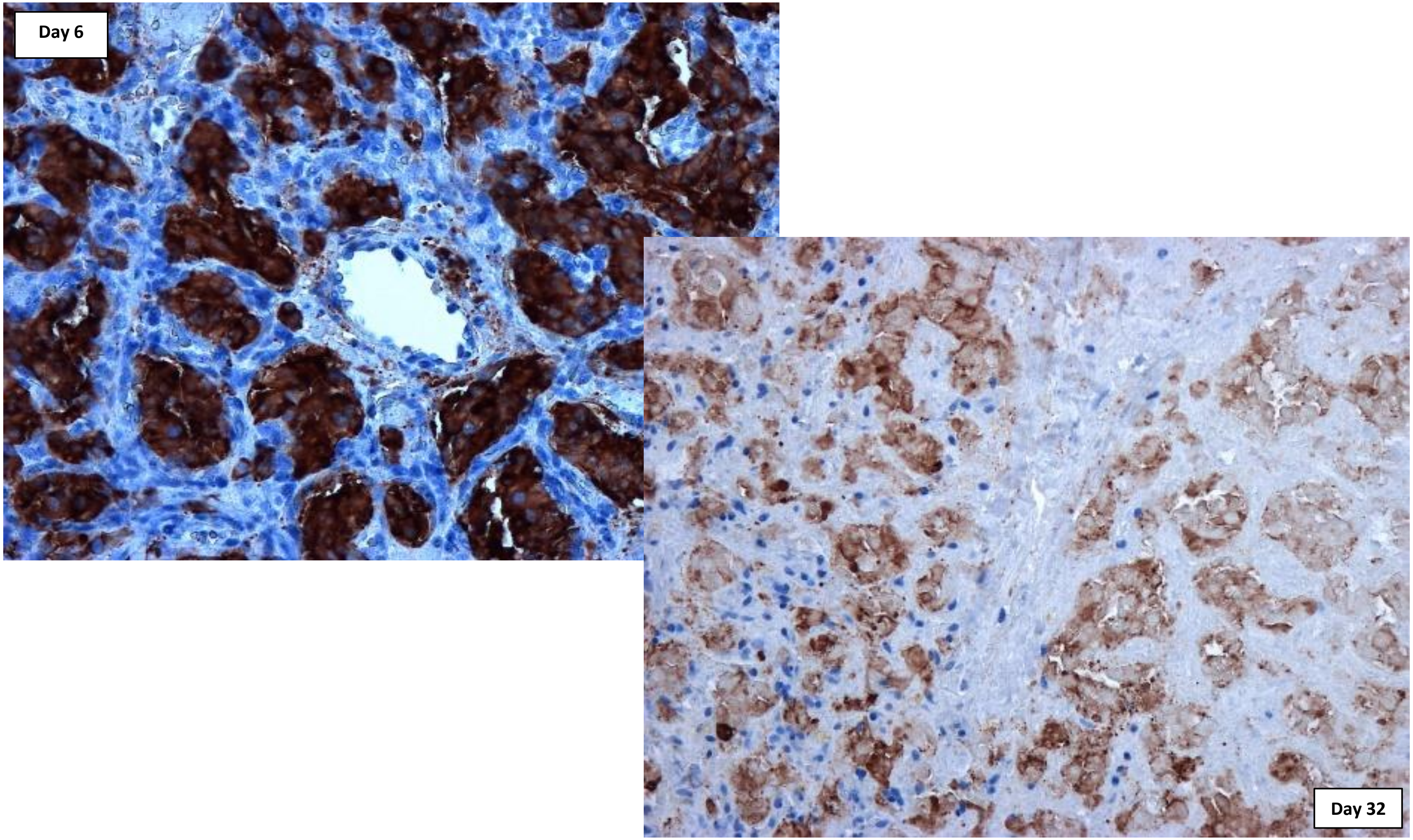

S2.4 Fig. Tumour explant culture to determine cellularity of explants. [Tu44, CBT right, SDHD]. Tumour explants (minced tumour fragments) cultured for up to 32 days to assess cellularity and proportion of synaptophysin-positive cells ('chromaffin' tumour cells) remaining. Up to day 15, the intensity of synaptophysin staining in cells remained relatively strong, with only limited signs of a decline in cellularity/expression.

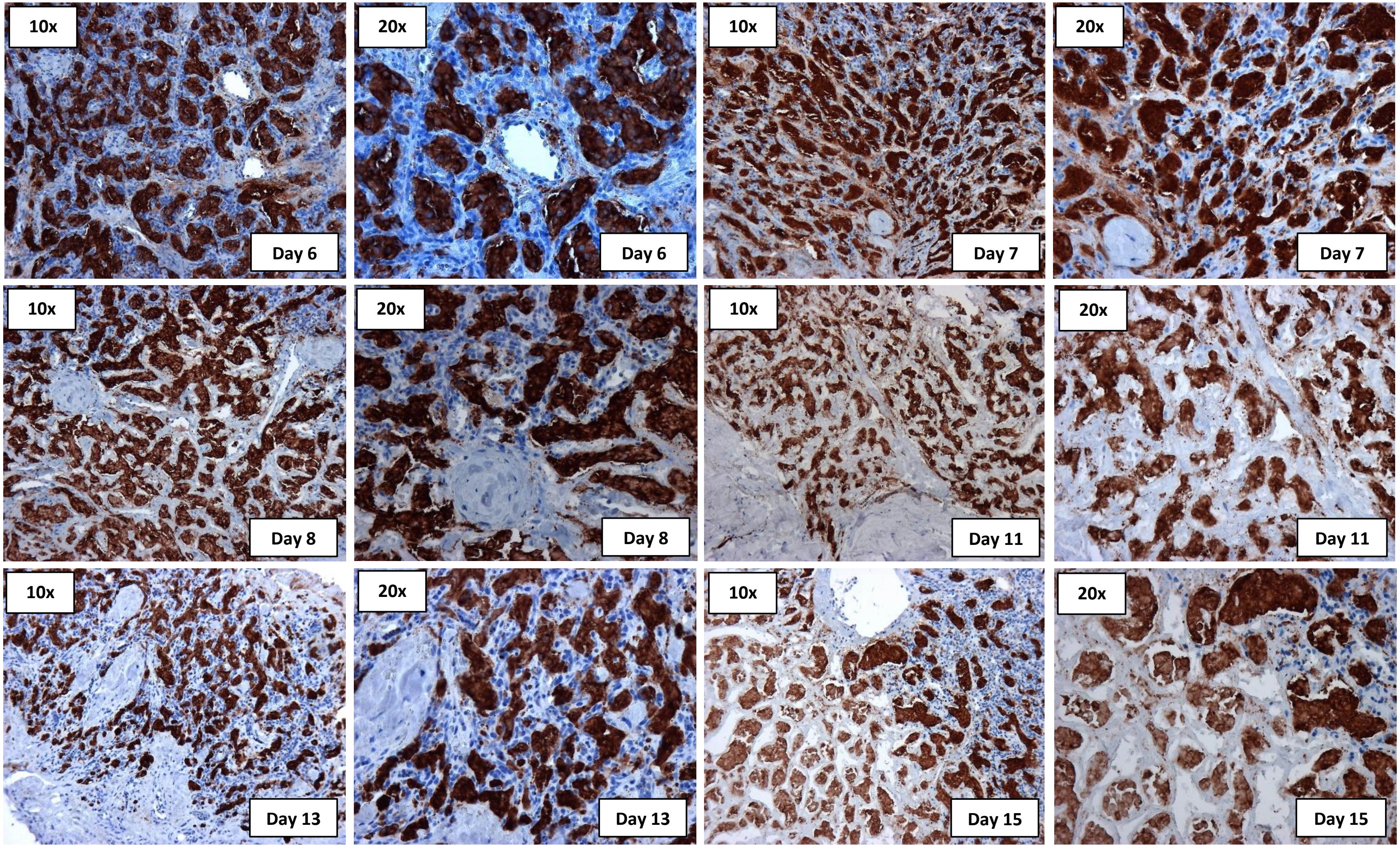

S2.5 Fig. Tumour explant culture to determine cellularity of explants. [Tu44, CBT right, SDHD].

Tumour explants (minced tumour fragments) cultured for up to 32 days to assess cellularity and proportion of synaptophysin-positive cells ('chromaffin' tumour cells) remaining. Around day 25 the tumour fragments begin to show clear signs of declining cellularity and declining expression of synaptophysin, with an apparent decrease in the intensity of synaptophysin staining in individual cells. This suggests that these cells were either in crisis or were already dead, with either a decline in the expression or stability of synaptophysin, or staining of residual protein in dying cells or amongst cell debris.

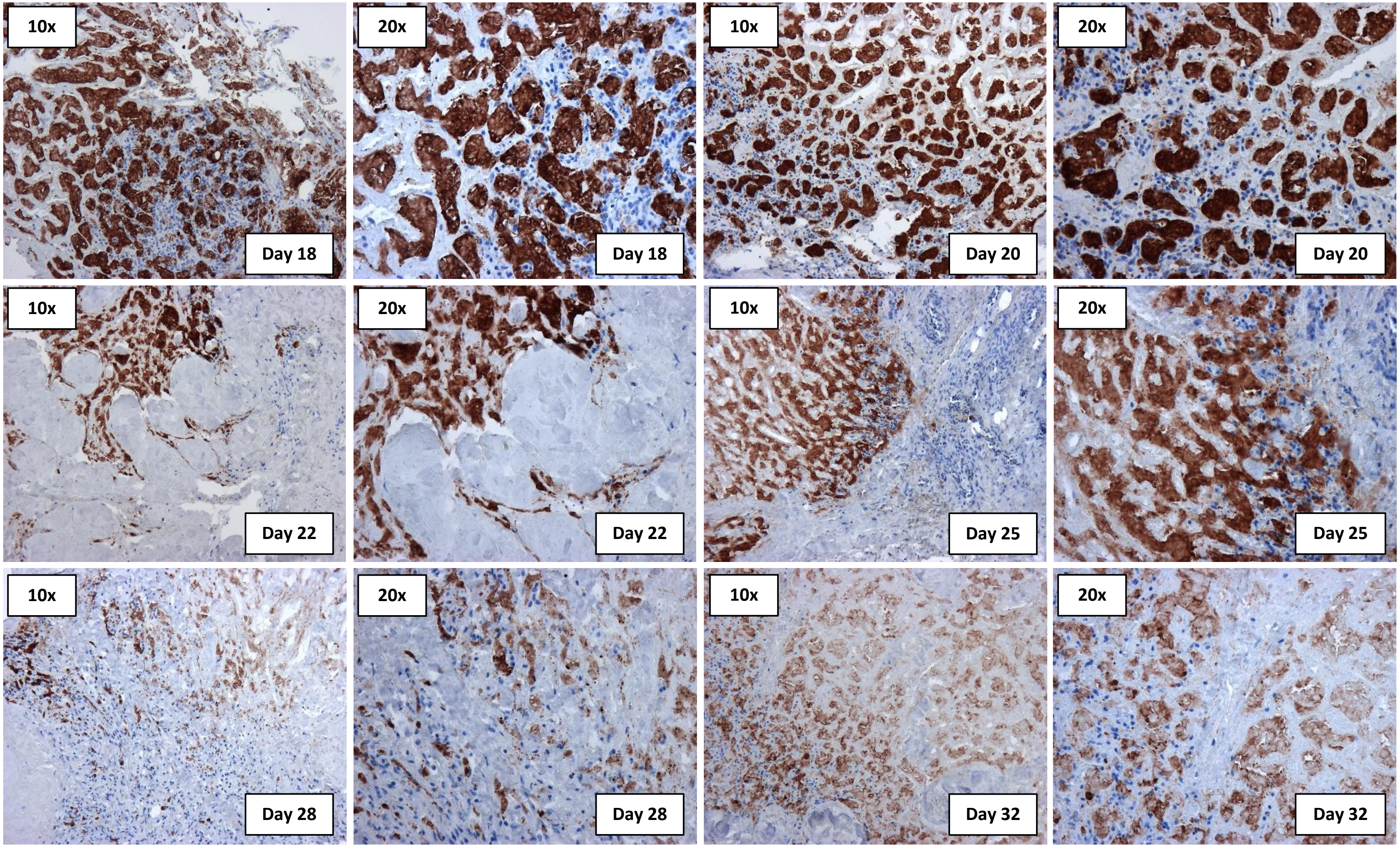

Supplement: S2 Fig — (PDF) [file pone.0274478.s002.pdf]
