# Supplementary material for: Long-term in vitro 2D-culture of SDHB and SDHD-related human paragangliomas and pheochromocytomas
Source: PLoS One. 2022 Sep 30;17(9):e0274478. doi: 10.1371/journal.pone.0274478 (PMC9524698; doi:10.1371/journal.pone.0274478)
Supplement: S3 Fig — (PDF) [file pone.0274478.s003.pdf]

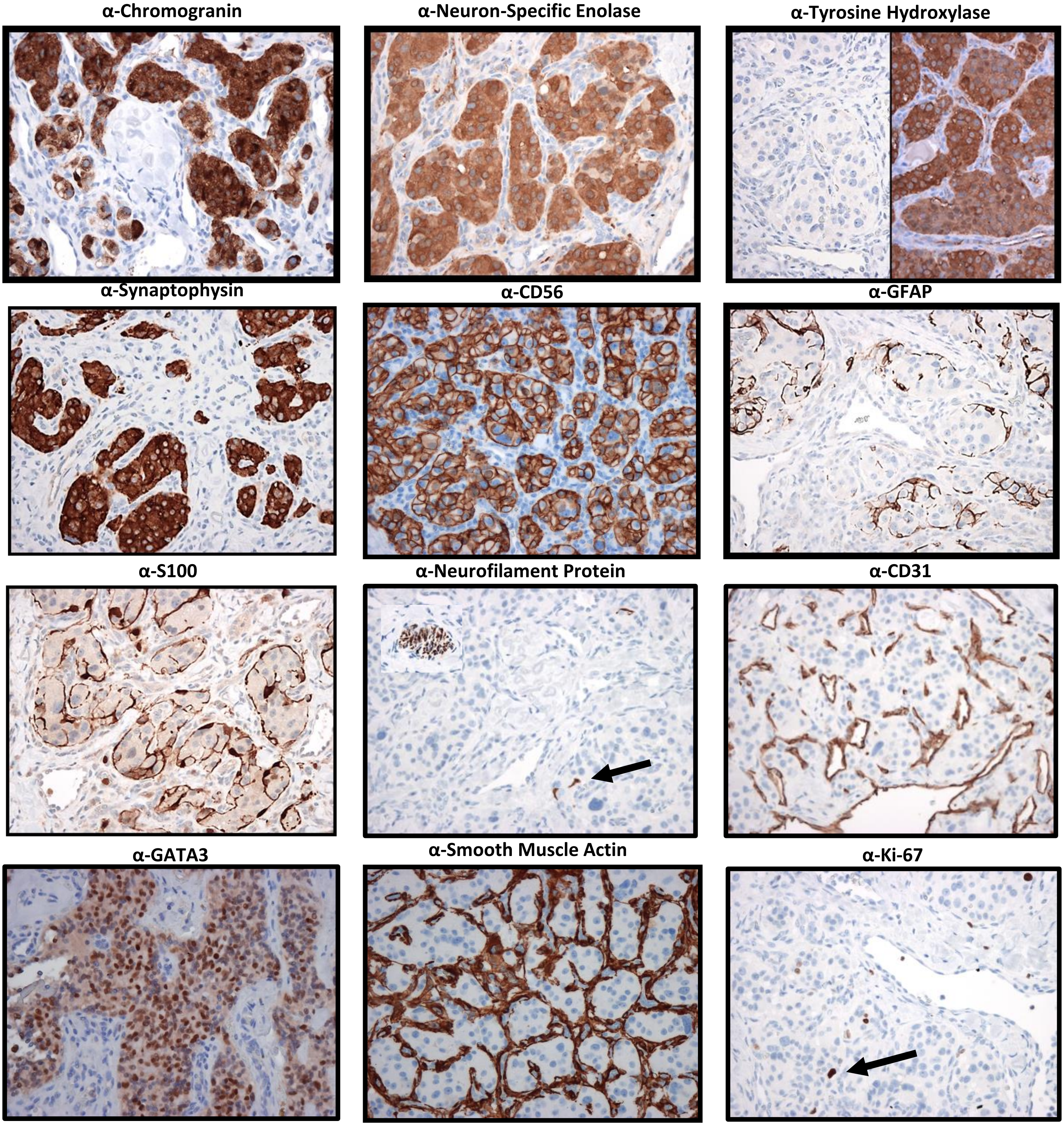

α-Chromogranin
α-Neuron-Specific Enolase
α-Tyrosine Hydroxylase
α-Synaptophysin
α-CD56
α-GFAP
α-S100
α-Neurofilament Protein
α-CD31
α-GATA3
α-Smooth Muscle Actin
α-Ki-67

S3 Fig. Antibody stainings of formalin-fixed paraffin-embedded (FFPE) PPGL tumors. Chromogranin A, neuron-specific enolase, tyrosine hydroxylase and synaptophysin staining are all commonly used markers for the chromaffin cell component of these tumors. However, as illustrated by two distinct tumors, many HNPGL cultures are negative for tyrosine hydroxylase, so this marker is not generally reliable. CD56 (NCAM) is a cell surface marker for chromaffin cells and is found in most PPGLs, showing the distinct staining pattern expected of a cell surface protein. S100 and GFAP are classic markers for sustentacular cells in PPGLs. GFAP is generally expressed more sporadically but in the same cells as S100.

Neurofilament protein stains nerve fibers and sparse individual neurons in PPGL tumors. CD31 stains endothelial cells and underscores the extensive vascularity of PPGLs. GATA3 is a transcription factor that shows specific but highly variable nuclear expression amongst cells in the cell nest region of PPGLs, most likely chromaffin cells. The cytoskeleton protein smooth muscle actin (SMA) stains the fibroblast component of tumors. Ki-67, a protein expressed in proliferating cells, is expressed only very sporadically in PPGL tumors, illustrating the relatively indolent nature of these tumors.
