# Supplementary material for: Long-term in vitro 2D-culture of SDHB and SDHD-related human paragangliomas and pheochromocytomas
Source: PLoS One. 2022 Sep 30;17(9):e0274478. doi: 10.1371/journal.pone.0274478 (PMC9524698; doi:10.1371/journal.pone.0274478)
Supplement: S4 Fig — (PDF) [file pone.0274478.s004.pdf]

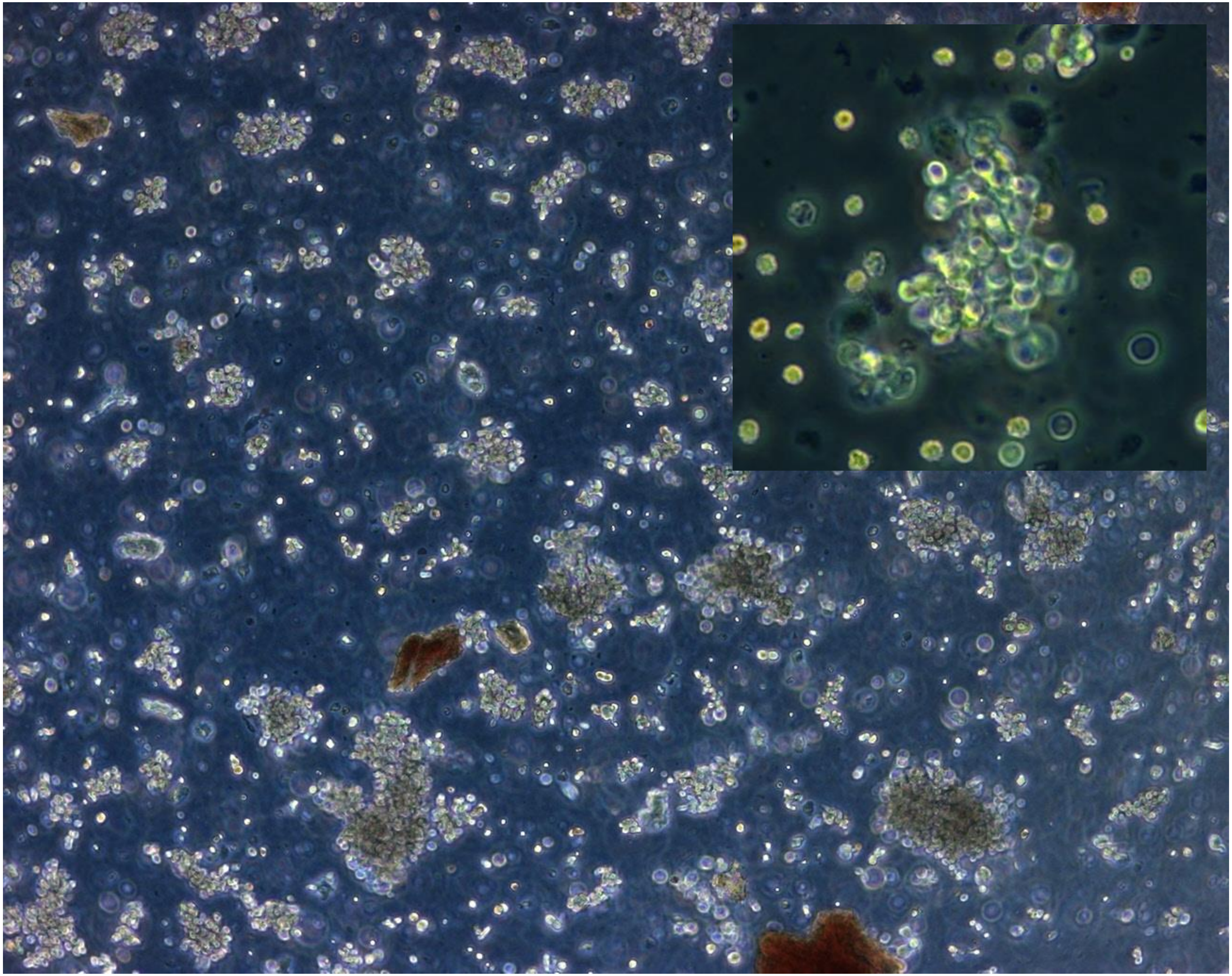

S3.1 Fig. Digestion of a carotid body tumour with collagenase-dispase followed by collagenase B. The main image (phase contrast 10x obj.) is fairly typical and shows clumps of chief (chromaffin) cells of variable size intermingled with cell debris and erythrocytes. The inset (phase contrast 40x obj.) shows a single clump of chromaffin cells, surrounded by erythrocytes.

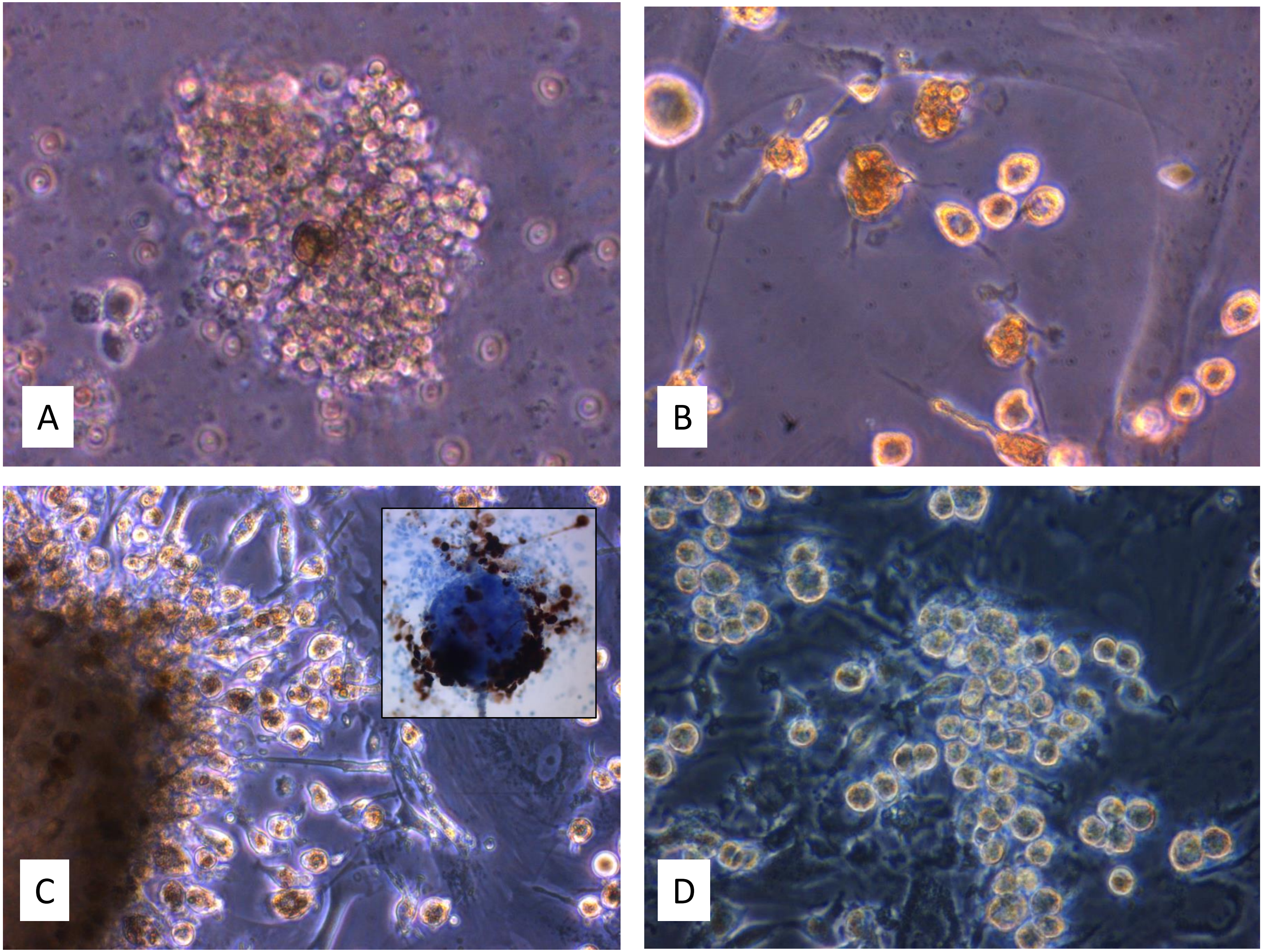
A
B
C
D

S3.2 Fig. Enzyme-mediated dissociation of paraganglioma tissue. Immediately following enzyme-mediated dissociation, paraganglioma tissue consists of single cells, small clumps of a few dozen cells and larger fragments of hundreds of loosely associated cells, as seen in A (Tu46_3 days_SF _20x obj.). Small cell clumps and single cells, maintaining a rounded morphology, generally disappear quite rapidly from culture (B, Tu46_17 days_5% FBS_40x obj.) unless securely anchored to a cellular substrate. Some cells take on a flattened, extended morphology and then appear to persist somewhat longer in culture than rounded, only semi-adherent cells. Larger cell clumps are a common feature of paraganglioma cultures, appear to be anchored by fibroblast-like cells to plastic substrate and often 'shed' cells with a chromaffin cell morphology and expressing synaptophysin during the first few weeks of culture (C, Tu42_35 days_5% FBS_20x obj.; inset, Tu44_1 day_20x obj.). These cells are often weakly adherent to either plastic or a fibroblast underlayer and can often be harvested by vigorous pipetting and re-cultured (D, Tu51_58 days _1% FBS_40x). If the aim is to remove all fibroblasts, this process must be repeated several times and the culture evaluated for cells of fibroblast morphology. However, removing fibroblasts doesn't assist the cell growth of chromaffin cells and may lead to loss of poorly adherent cells.

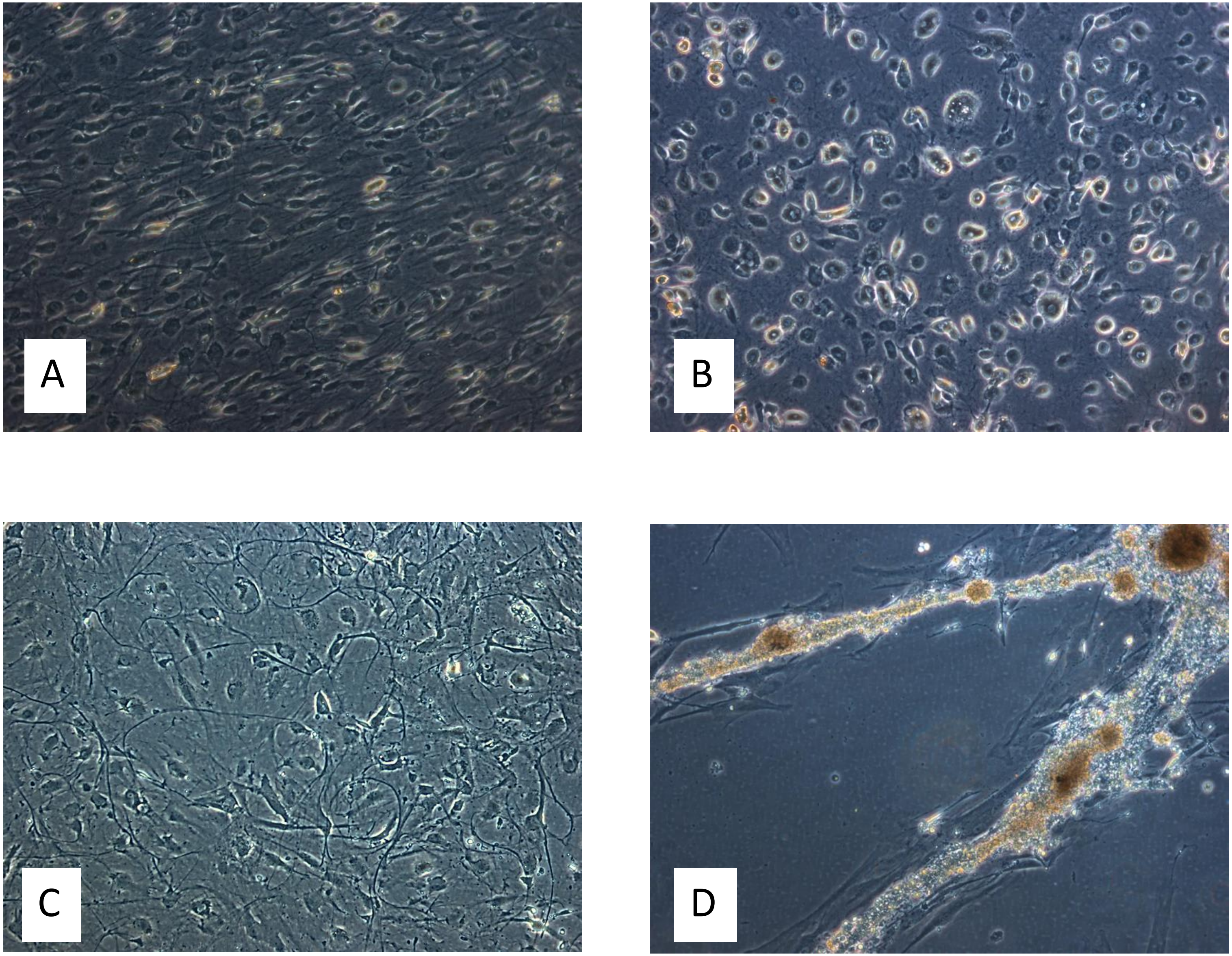
A
B
C
D

S3.3 Fig. Long-term PPGL cultures. Many long-term PPGL cultures eventually develop a uniform flattened sheet-like appearance with prominent central cell bodies, as seen in A and B (Tu2_36 months 5% FBS_10x obj.; Tu28_28 months_5% FBS_10x obj.). Other cultures show more specific structure, including cells with long processes of neuronal appearance (C, Tu16_23 months_5% FBS_10x obj.) or directional, multicellular processes that may even be macroscopically visible (D, Tu26_17 months_SF_10x obj.).

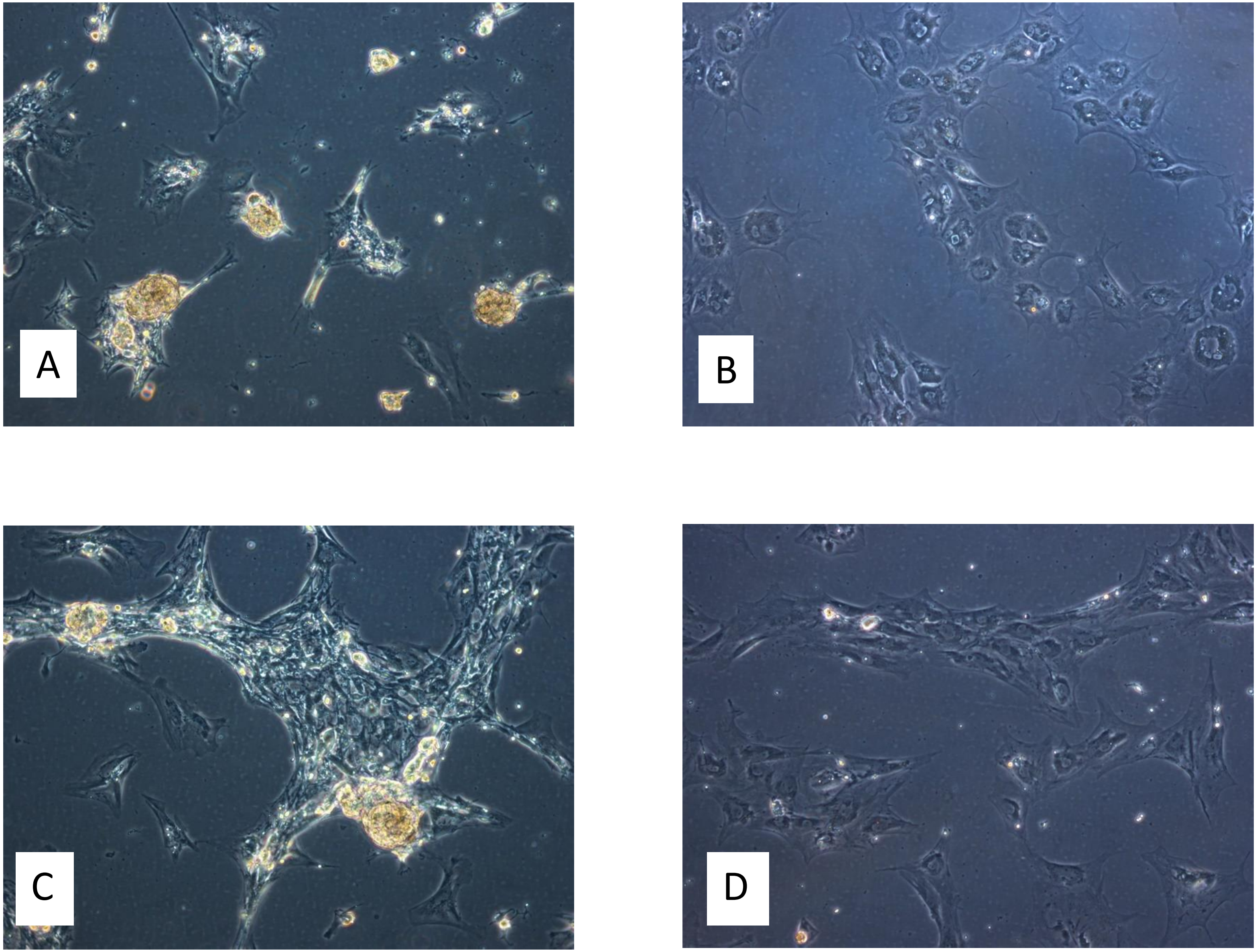
A
B
C
D

S3.4 Fig. Small and large cell masses in paraganglioma cultures. Other long-term PPGL cultures develop dispersed small and large cell masses, often surrounded by flattened cells that show limited cell spreading and a prominent main cell body, as seen in A and B (Tu26_7 months_5% FBS_10x obj.; Tu32_15 months_1% FBS_10x obj.). Other cultures show greater structure, including flattened cell masses containing numerous cells (C, Tu42_36 months_5% FBS_10x obj.) or extended structures consisting of relatively few cells (D, Tu43_8 months_1% FBS_10x obj.).

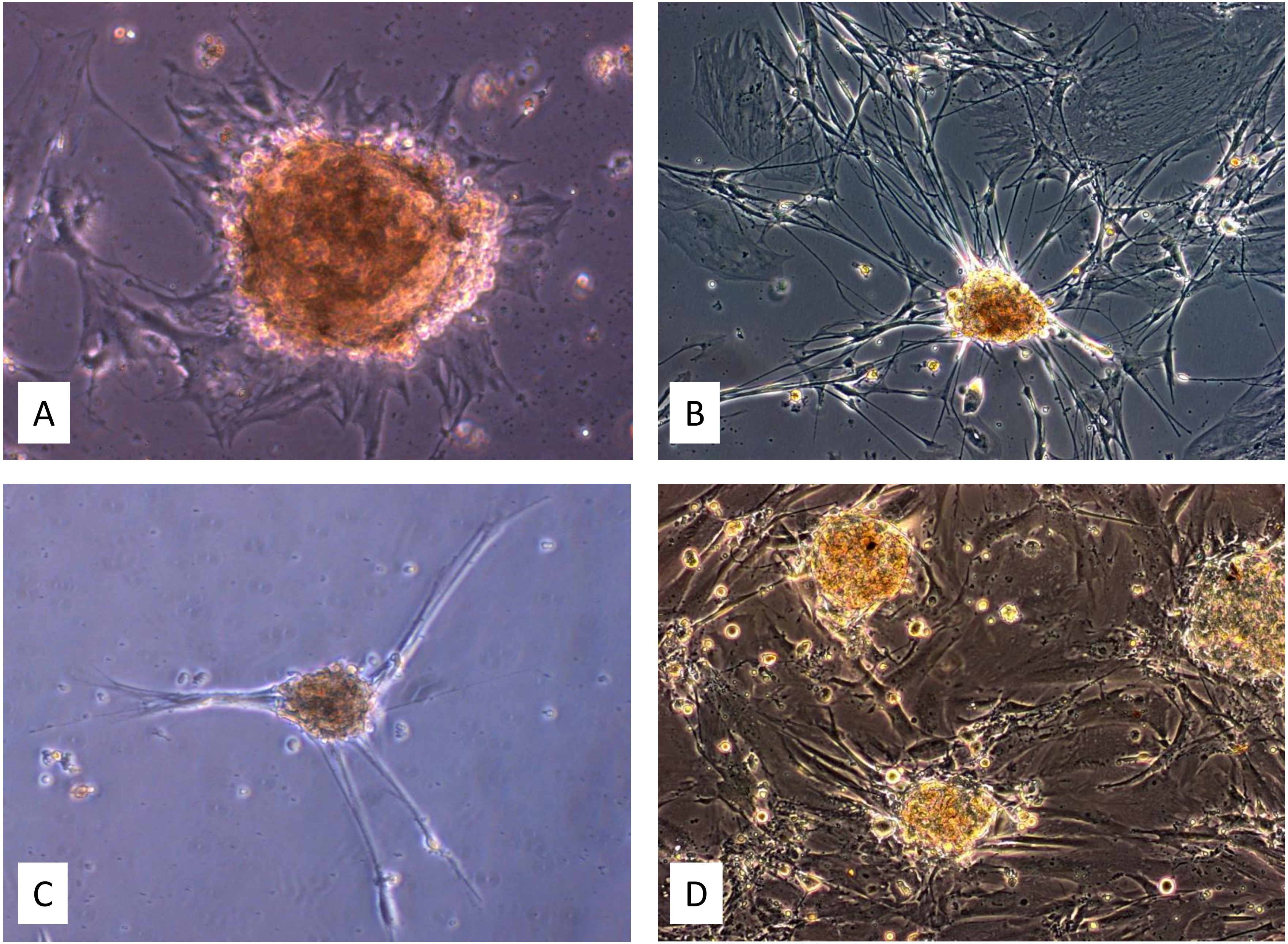
A
B
C
D

S3.5 Fig. Typical appearance of 'cell masses' in paraganglioma tumour cultures. Images A-C are early cultures (days 16, 23 and 31, respectively) which appear to have formed a cell mass anchored on cells with either a fibroblast-like aspect (A, Tu42_day 16_1% FBS_20x obj.) or a neuronal aspect (B, Tu11_23 days_5% FBS_20x; C, Tu39_31 days_SF_10x; most likely either neuronal cells or the sustentacular cells typically found in non-metastatic tumours). Large, flattened fibroblasts can also be seen in image B. Image D shows a typical late culture (Tu7_18 months_5% FBS_20x obj.), with a cell mass formed on a layer of cells with fibroblast-like morphology.

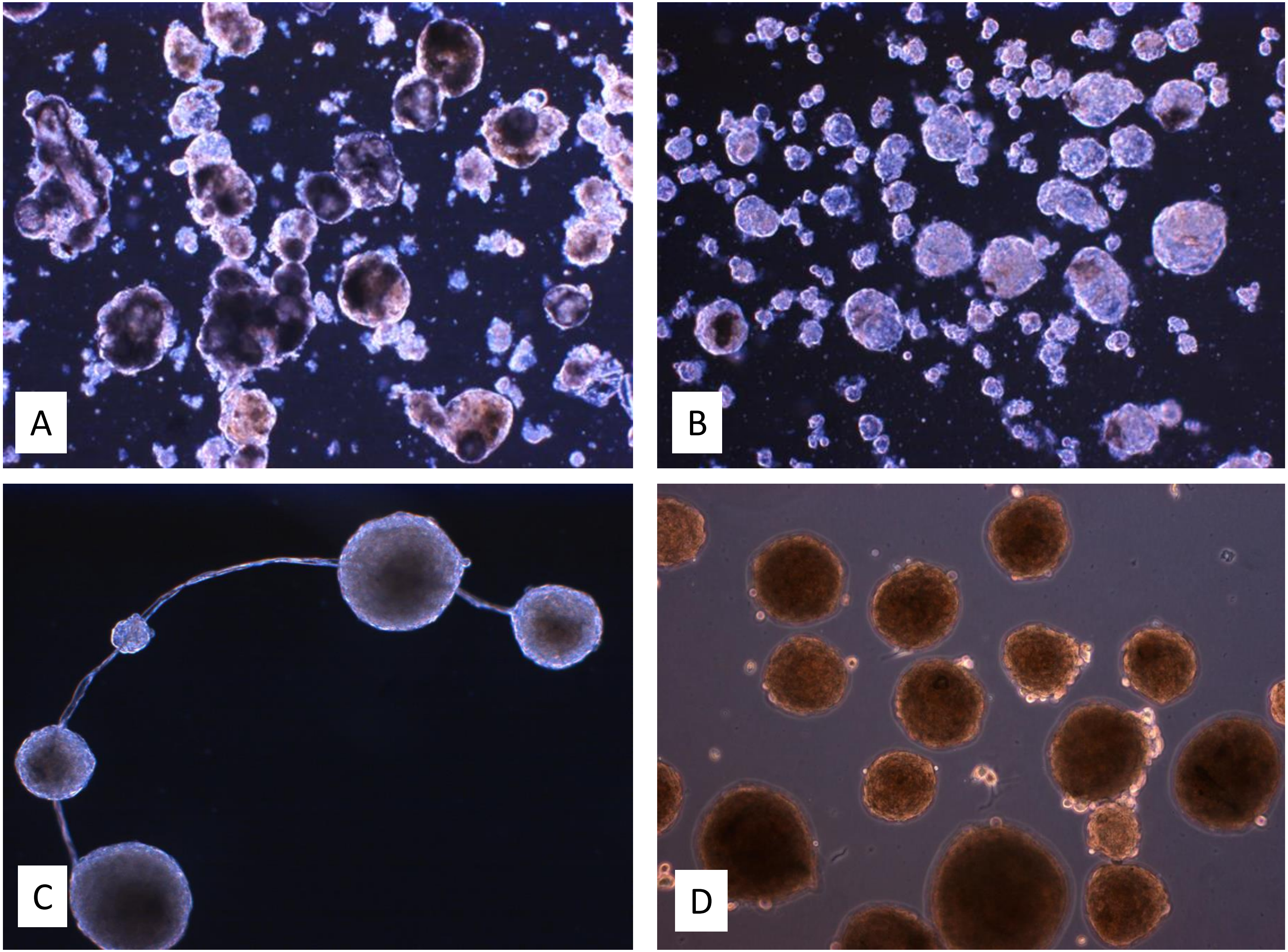
A
B
C
D

S3.6 Fig. Discrete cell masses. Tumour cultures occasionally show discrete cell masses, either following culture on low-adhesion plates (A, Tu26_10 months_PC12 Med_5x obj.; B, Tu34_9 months_1% FBS_5x obj.; C, Tu44_3 months _SF_5x obj.) or on standard plastic (D, Tu51_78 days_SF_10x obj.). Tumour tissue cultured in SF medium spontaneously produced rounded, free-floating cell masses on several occasions without the use of low-adhesion plates. Free-floating cell masses are otherwise rare, as the cell masses that commonly form in paraganglioma cell cultures are generally anchored by fibroblast-like cells.

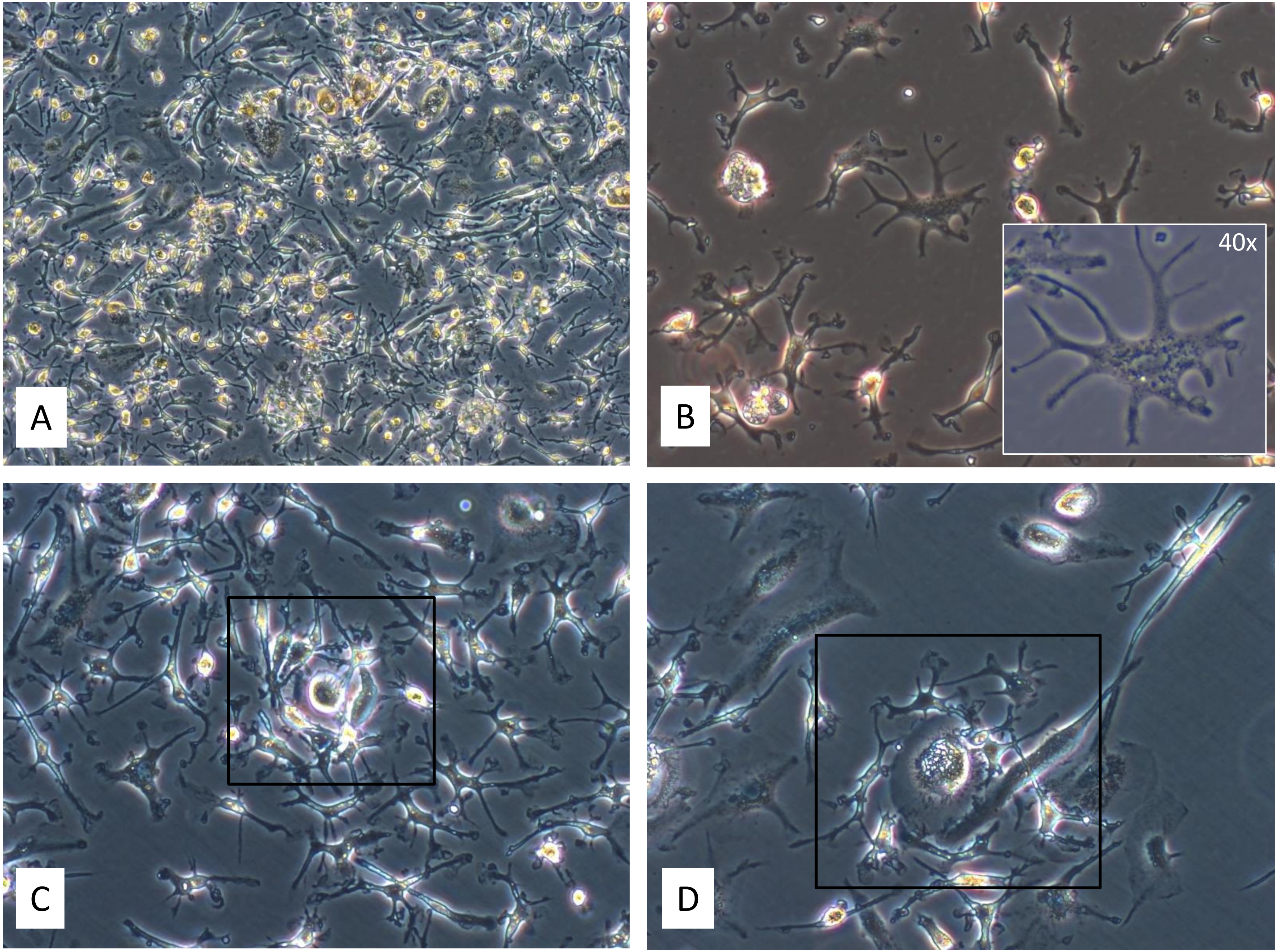
A
B
40x
C
D

S3.7 Fig. Culture of a non-SDHx tumour. Tumour culture 23, a paraganglioma without a mutation in any commonly-mutated gene, consisted of many cells exhibiting short, eccentric branching of cell processes, but also occasionally showing extensive neuronal-like processes, which are presumably differentiating chromaffin cells and/or sustentacular cells (A & B, Tu23_20 days_5% FBS_10x, 20x (and 40x obj. inset), resp.). Another occasional feature of other PGL cultures but particularly visible in this culture was the accumulation of a number of these cells around a single, flattened cell, as clearly visible in images C and D (boxes; 20x obj. and 40x obj., resp.). Tumour 23 showed clear visual evidence for cellular proliferation for several weeks, followed by slow thinning of cell number and eventual predomination of fibroblast-like cells. Further isolation of chromaffin cells using differential adhesion did not appear to lead to extended survival of these cells.

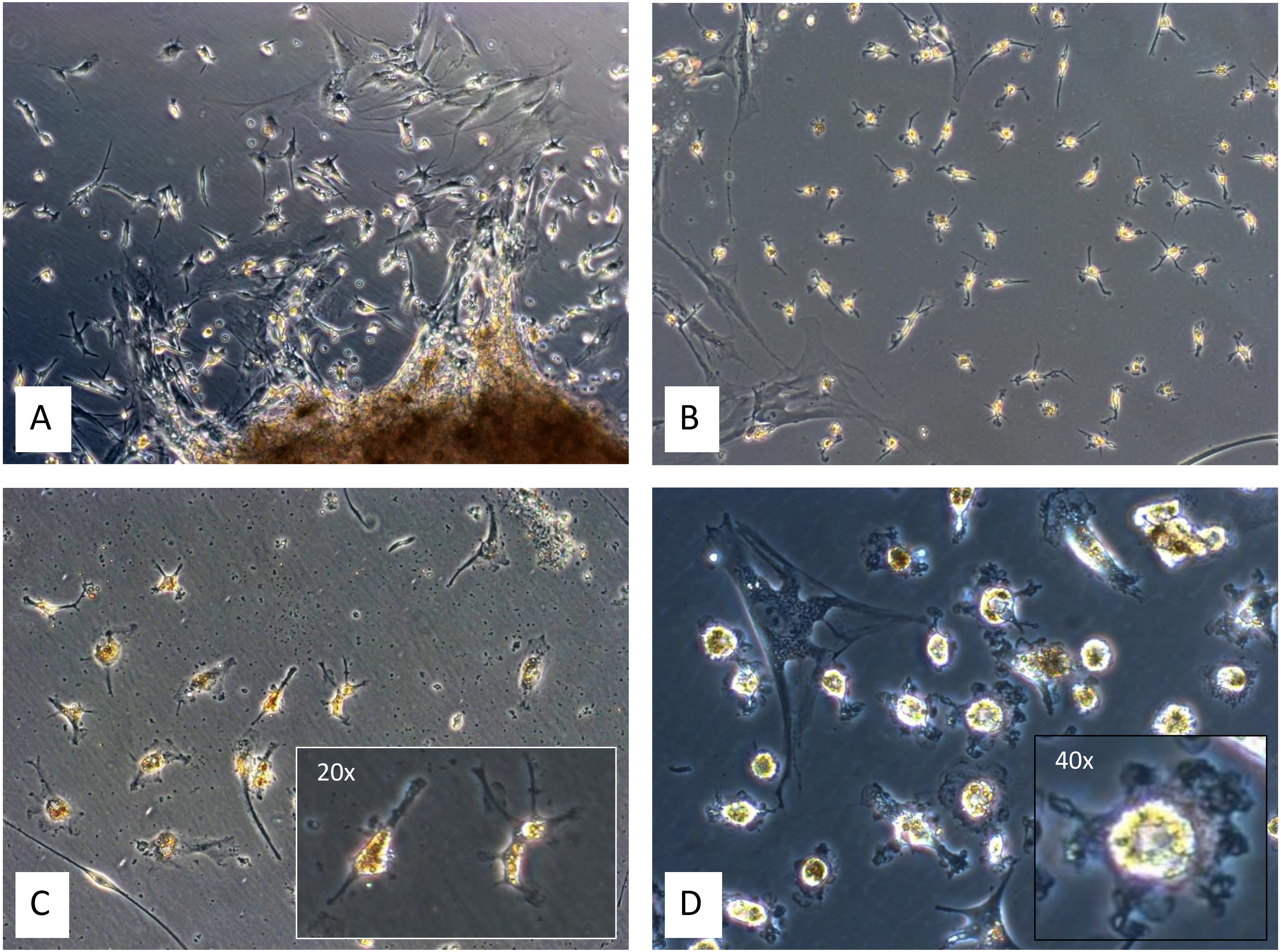
A
B
C
20x
D
40x

S3.8 Fig. Cell masses often shed cells. A cell mass is seen shedding cells with differentiated chromaffin-like morphology (A, Tu16_4 days_5% FBS_5x obj.), which adhere and spread on a plastic surface. In other cultures, cells may show a rounded, semi-adherent morphology with extensive filopodia (B, Tu11_14 days_5% FBS_5x obj.; C, Tu28_15 days_1% FBS_10x) that may attach either vigorously or poorly (D, Tu28_19 days_IZAL_20x obj.) to a plastic substrate. Cells with chromaffin-like morphology often seem to initially poorly adhere to both plastic and a fibroblast cell layer and may therefore be lost from culture. When shed onto a confluent layer of mature fibroblasts chief cells often maintain a rounded, semi-adherent aspect and can often be dislodged by shaking a culture flask or vigorous pipetting, although other cells may require trypinsinization.

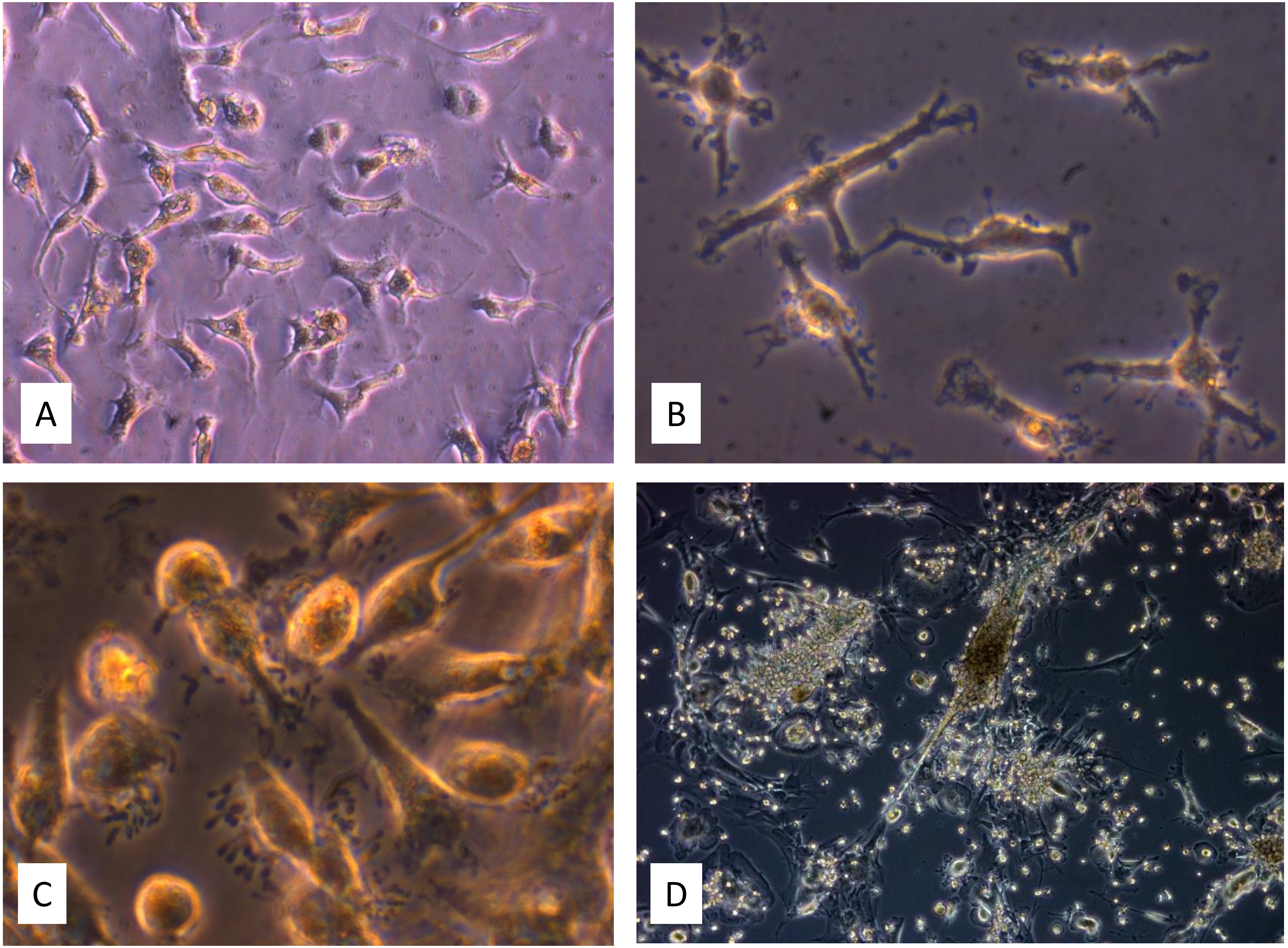
A
B
C
D

S3.9 Fig. Chief cells. Cells with a chief cell morphology tend to be more clearly visible and may persist for months in cultures that are not become quickly dominated by fibroblasts, as seen in A and B (A, Tu46_22 days_1% FBS_20x obj.; B, Tu47_7 weeks_SF_40x obj.). On the other hand, single cells and cell clumps with chromaffin morphology often associate with cells of fibroblast morphology (C, Tu47_8 weeks_1% FBS_40x obj.; D, Tu57_12 weeks_1% FBS _10x obj.) and can persist on these substrate cells for years in culture.

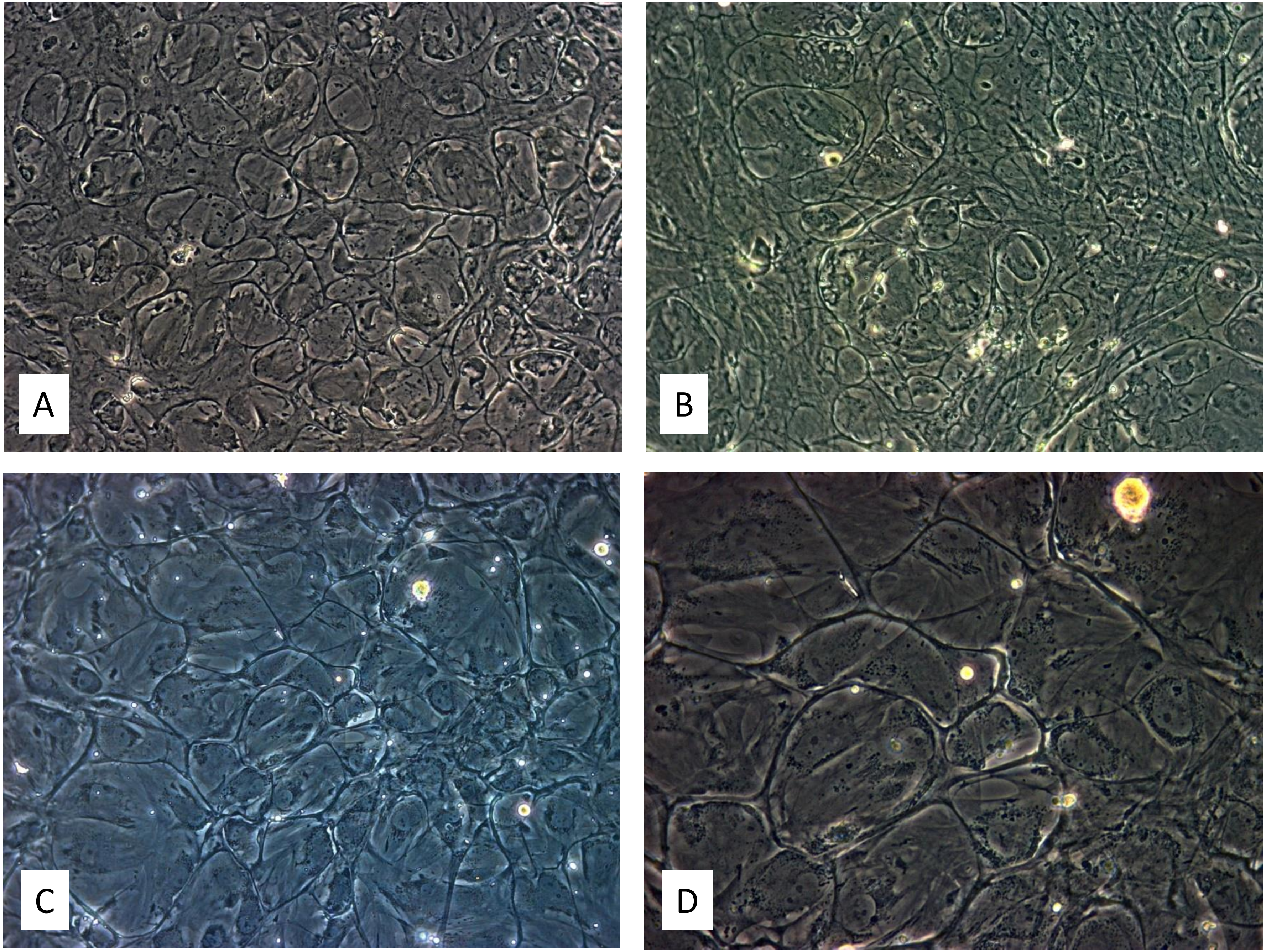
A
B
C
D

S3.10 Fig. 'Frustrated zellballen'. Long-term cultures occasionally develop areas that show roughly circular lattice structures (A, Tu 11_4 months_5% FBS_10x obj. & B, Tu23_3 months_5% FBS_10x obj.) that may represent sustentacular cells forming networks reminiscent of those found in tumours. However, as immunocytochemical analysis suggests that sustentacular cells show poor survival in culture (<4 weeks), these structures more likely represent connective tissue cells specific to PPGLs, and perhaps suggest that these cells have self-organizing capabilities specific to this tissue. Due the delicacy of these structures and the many months required before they develop (C, Tu49 _5 months_1% FBS_20x obj. & D, Tu49 _5 months_1% FBS_40x obj.), it was not possible to carry out immunocytochemical analysis.

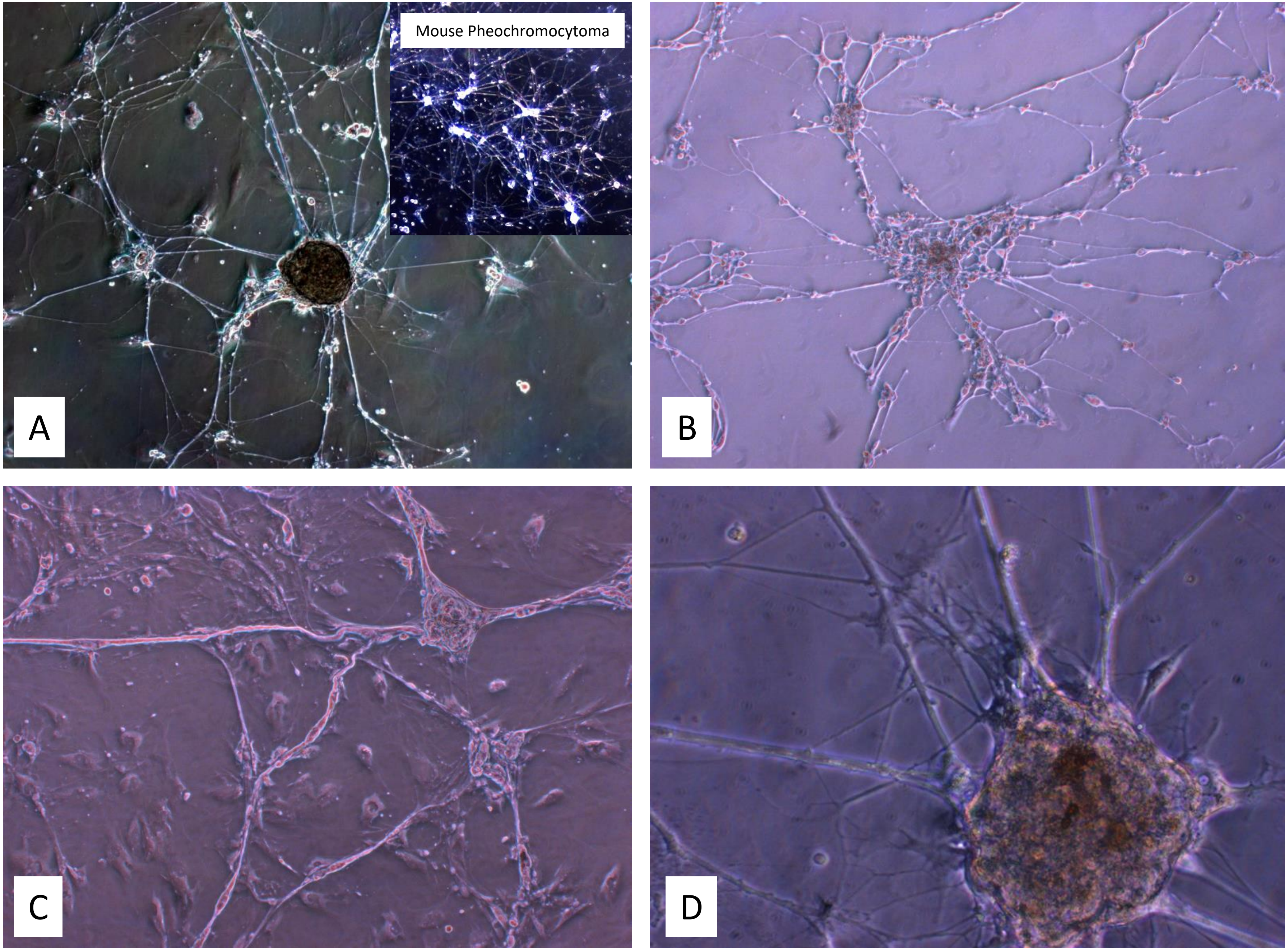

Mouse Pheochromocytoma
A
B
C
D

S3.11 Fig. Networks of processes. Paraganglioma cell cultures occasionally develop extensive networks of processes, usually but not always after an extended period in culture. These networks may become macroscopically visible in cell culture flasks and usually interconnect discrete cell masses (A, Tu21_4 months_5% FBS_5x obj.; B, Tu28_5 months_SF_5x obj.; C, Tu25_9 months_5% FBS_5x obj.; D, Tu42_2 months_5% FBS_20x obj.). It is unclear which cells are represented as the delicacy of the networks precludes analysis using a chamber slide approach. However, it is possible that these structures may be of chromaffin cell origin, and similar structures were also observed in a culture of primary mouse adrenal pheochromocytoma cells (inset A, detail).

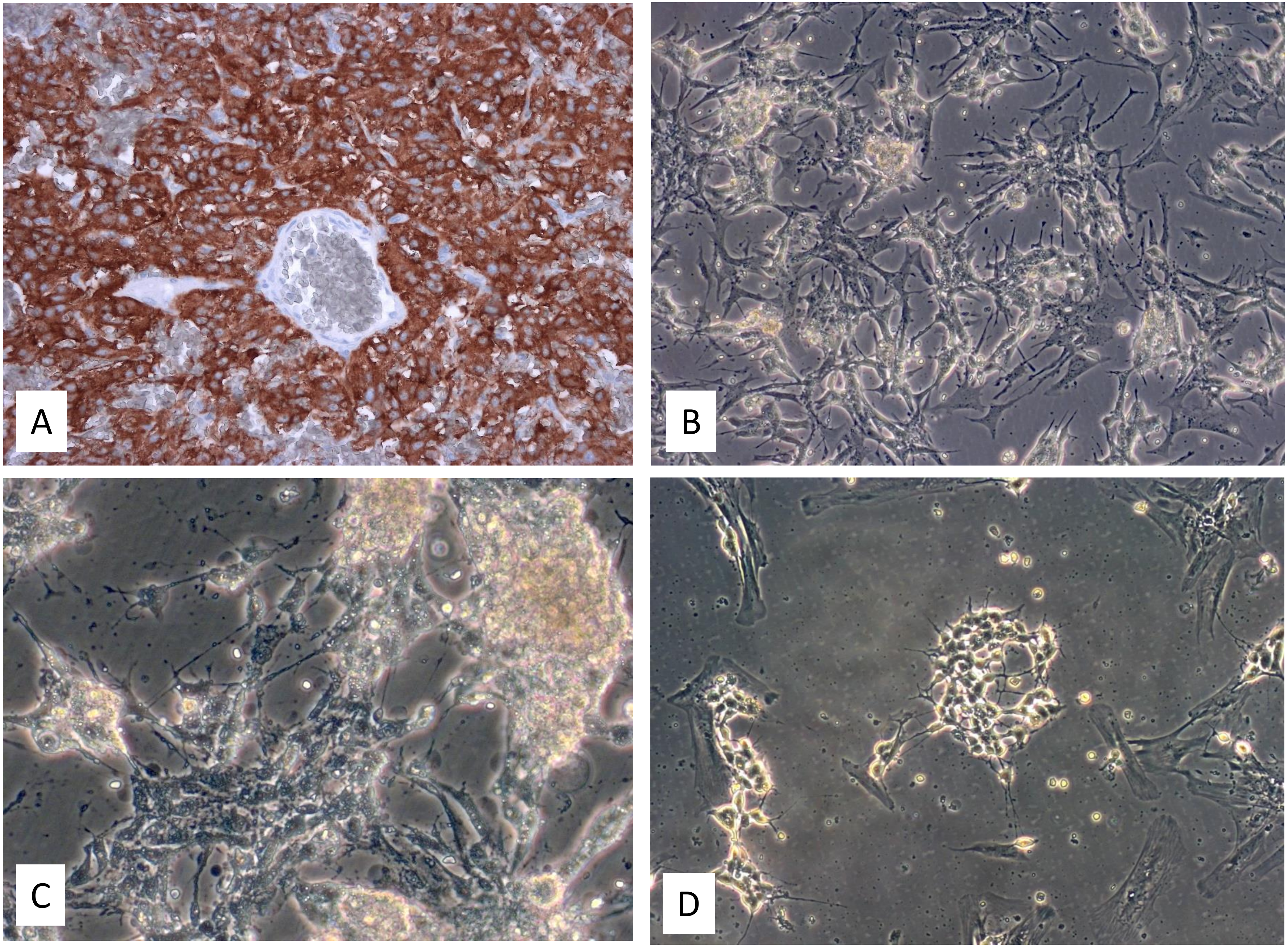
A
B
C
D

S3.12 Fig. Culture of a pheochromocytoma bone metastasis. The formalin-fixed tumor was stained with anti-synaptophysin (A, Tu26_10x obj.). Phase contrast images of cultures on day 6 (B, Tu26_6 days_5% FBS_10x obj.;C, Tu26_day 6_Med 3_20x obj.) and day 29 (D, Tu26_29 days_5% FBS_10x obj.) reveal cells that exhibit a uniform differentiated chromaffin cell morphology, with short processes and limited cell spreading.

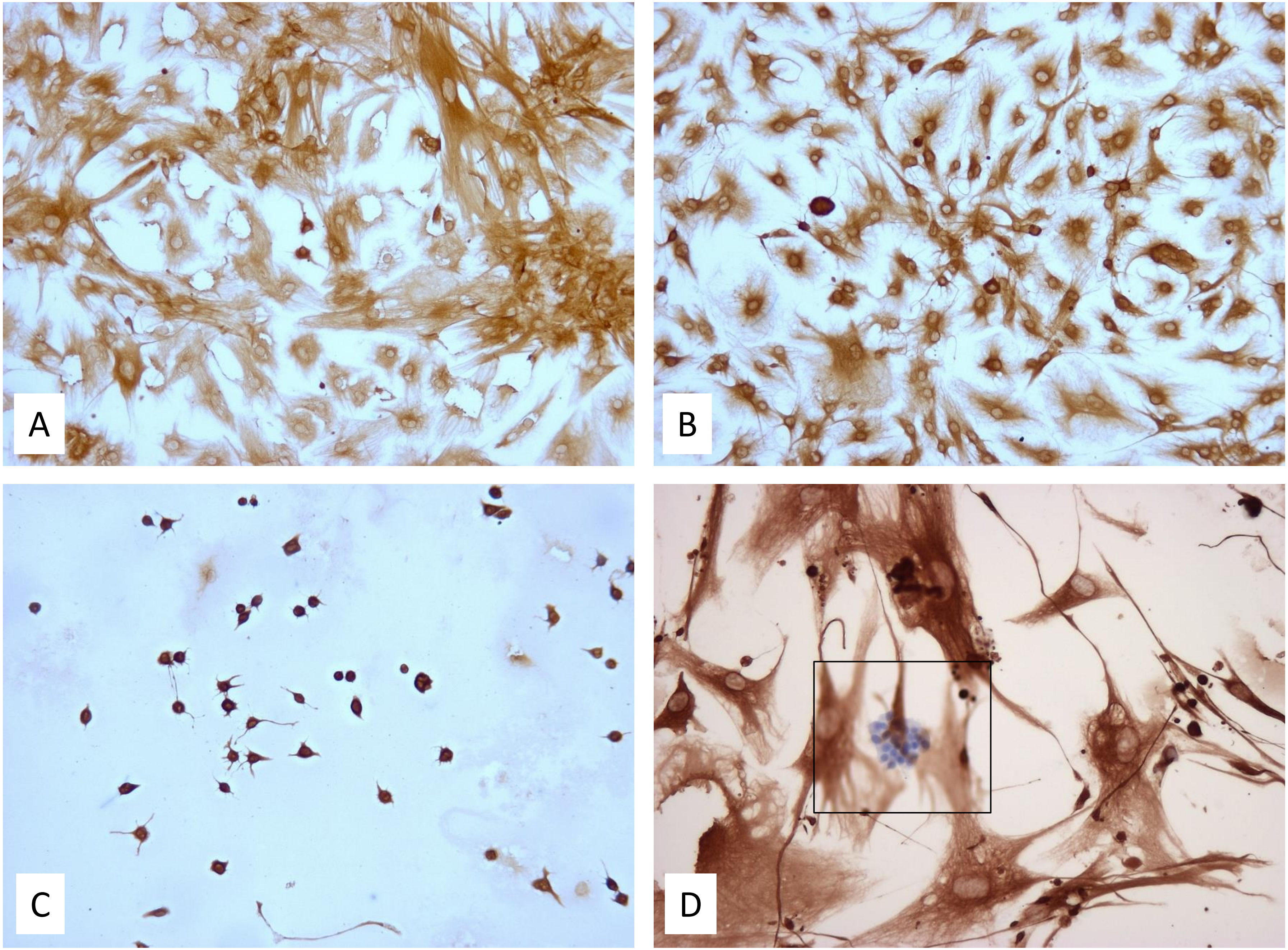
A
B
C
D

S3.13 Fig. Vimentin. Vimentin is a reliable control immunocytochemical stain in paraganglioma tumour cultures, aiding visualization of the overall morphology of positive-staining cells (A, Tu29_9 months_1% FBS_Vim_10x obj.; B, Tu29_9 months_5% FBS_Vim_10x obj.; C, Tu29_9 months_SF_Vim_10x obj.). Vimentin stains most cell types present in paraganglioma tumour cultures, including most cells of sustentacular and chromaffin morphology, but is not always positive in cells of undifferentiated chromaffin morphology (box, Tu-32_20 weeks_5% FBS_Vimentin_20x obj.).

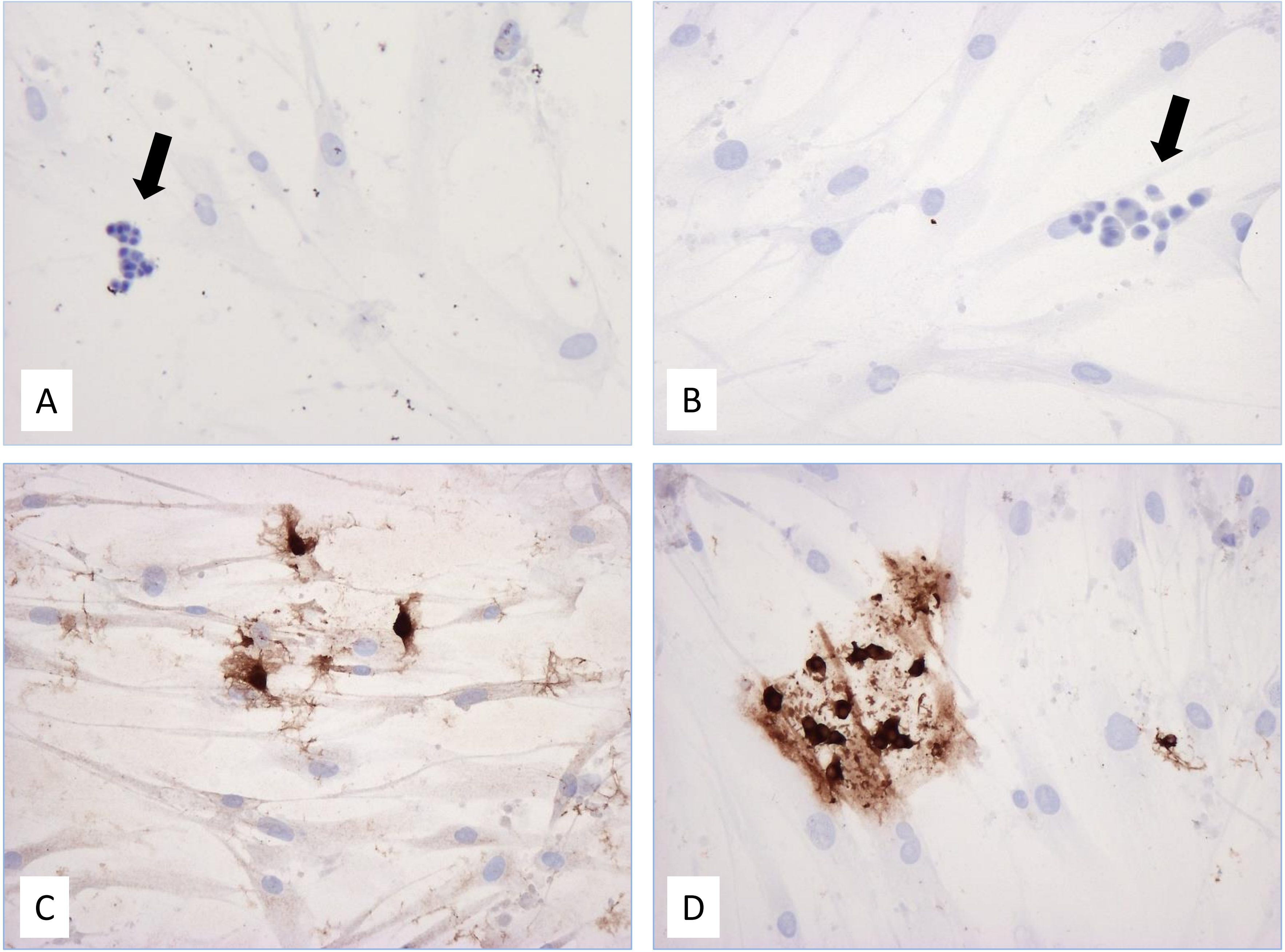
A
B
C
D

S3.14 Fig. Persistence of cells. Cells of undifferentiated chromaffin morphology persist in tumour cultures for long periods. They generally appear as compact cells characterized by sparse cytoplasm and small nuclei compared to large surrounding cells of fibroblast morphology and are often found in small clusters (A & B, stained with haematoxylin, arrows, Tu32_24 wks_5% FBS_20x obj.). Immunohistochemical staining with tyrosine hydroxylase (C) and synaptophysin (D) confirms the identity of these cells.

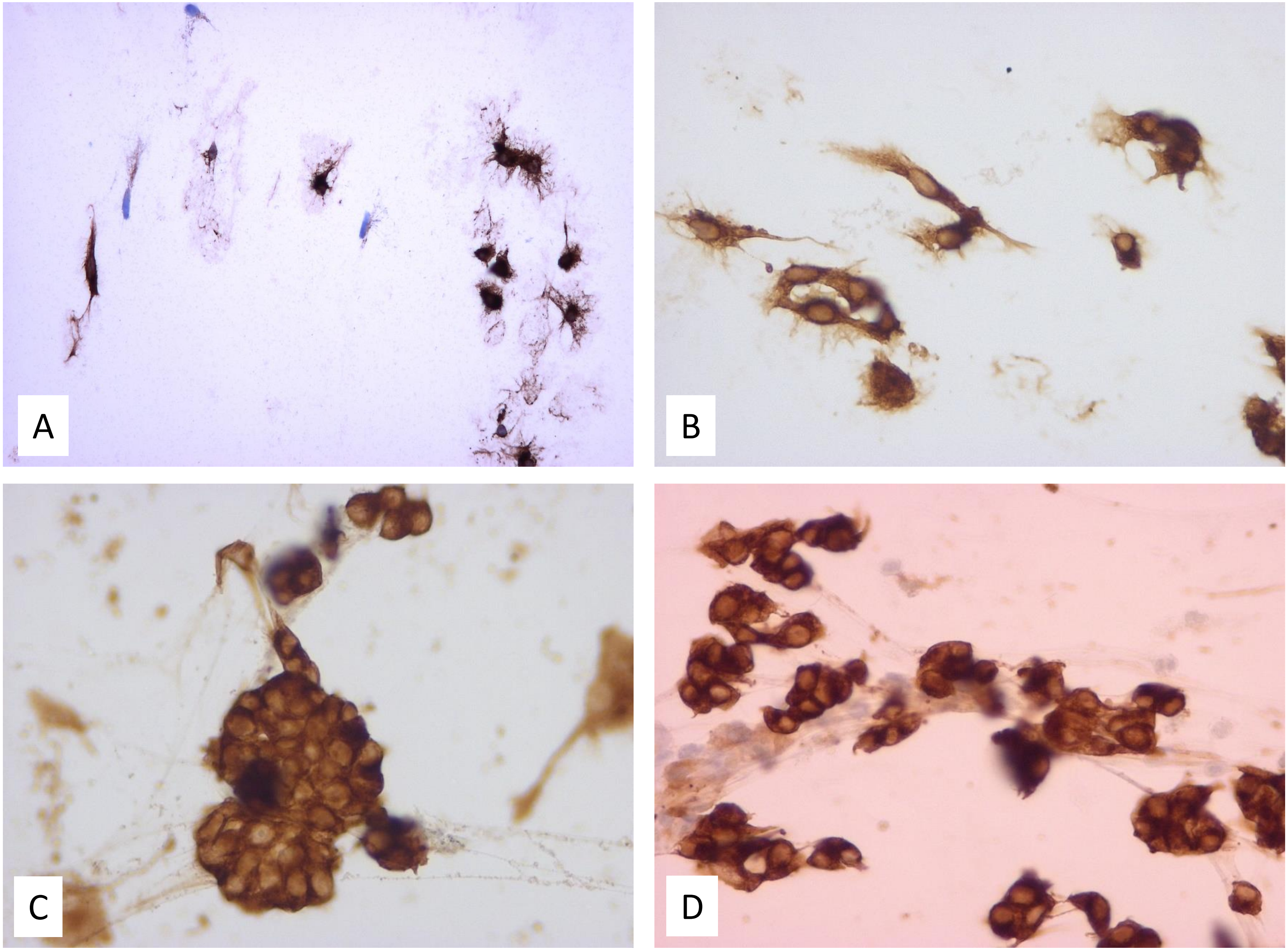
A
B
C
D

S3.15 Fig. Chromaffin cells often maintain a relatively undifferentiated morphology in tumour cultures. Immunohistochemical staining with synaptophysin confirms identity as chromaffin cells. These cells usually remain compact, show sparse cytoplasm and small nuclei compared to surrounding cells, and often cluster together, either attaching directly to plastic (A & B) or congregating on a prominent substrate (C & D). Cells often (A, Tu7_23 months_Syn_10x obj.) remain relatively compact and show little or no development of processes. Cells in low-serum medium either develop short processes (B, Tu43_4 weeks_1% FBS_Syn_20x obj.) or remain very compact (C, Tu42_4 weeks_5% FBS_Syn_40x obj.). Compact, undifferentiated cells often associate with a prominent substrate (D, Tu42_4 weeks_1% FBS_Syn_40x obj.).

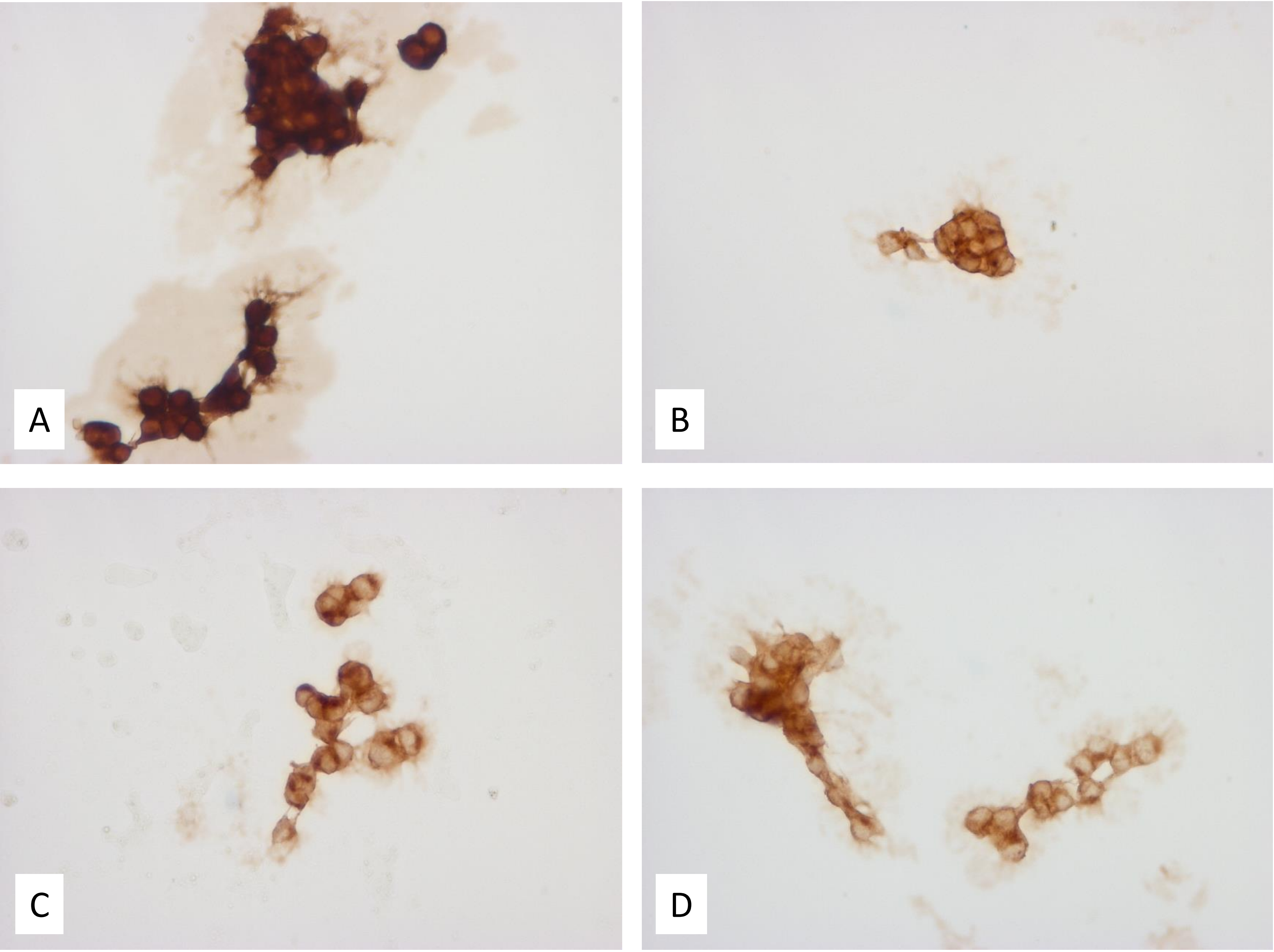
A
B
C
D

S3.16 Fig. Undifferentiated chromaffin cells. Further examples of relatively undifferentiated chromaffin cells in tumour culture adherent to plastic (A, Tu36_24 wks_TH_40x obj.; B-D, Tu36_24 wks_Syn_40x obj.), staining for either tyrosine hydroxylase (A) or synaptophysin, (B-D).

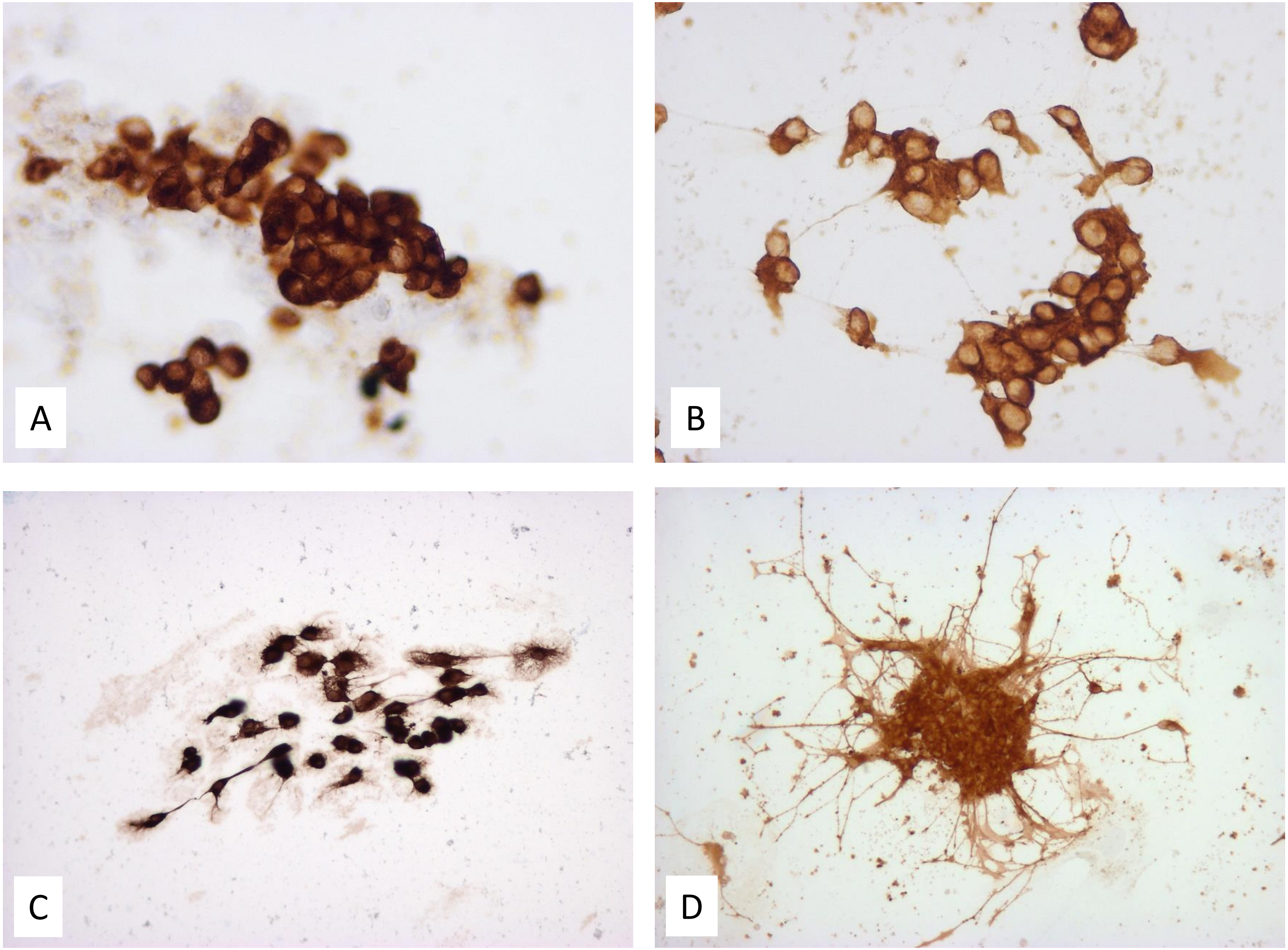
A
B
C
D

S3.17 Fig. Chromaffin cells show differing morphologies. Chromaffin cells show either a relatively undifferentiated morphology in tumour cultures (A & B, Tu42_4 weeks_5% FBS_Syn_40x obj.), stain intensely for synaptophysin, but may also develop a more differentiated morphology that includes short (C, Tum7_23 months_Syn_20x obj.) or more extended neuronal-like processes (D, Tum42_4 weeks_1%FBS_Syn_20x obj.). Relatively undifferentiated cells exhibit a compact morphology, are often present in small clusters, and show a relatively high nuclear-cytoplasmic ratio. Compact cells of undifferentiated or relatively undifferentiated morphology were the only Syn+ chromaffin cells found in long-term cultures. Although neuronal cells also express synaptophysin and staining with neurofilament protein (NFP) suggests that some neurons may persist in cultures, NFP-positive cells are generally sparse and thus seem an unlikely source for the cell agglomeration seen in D.

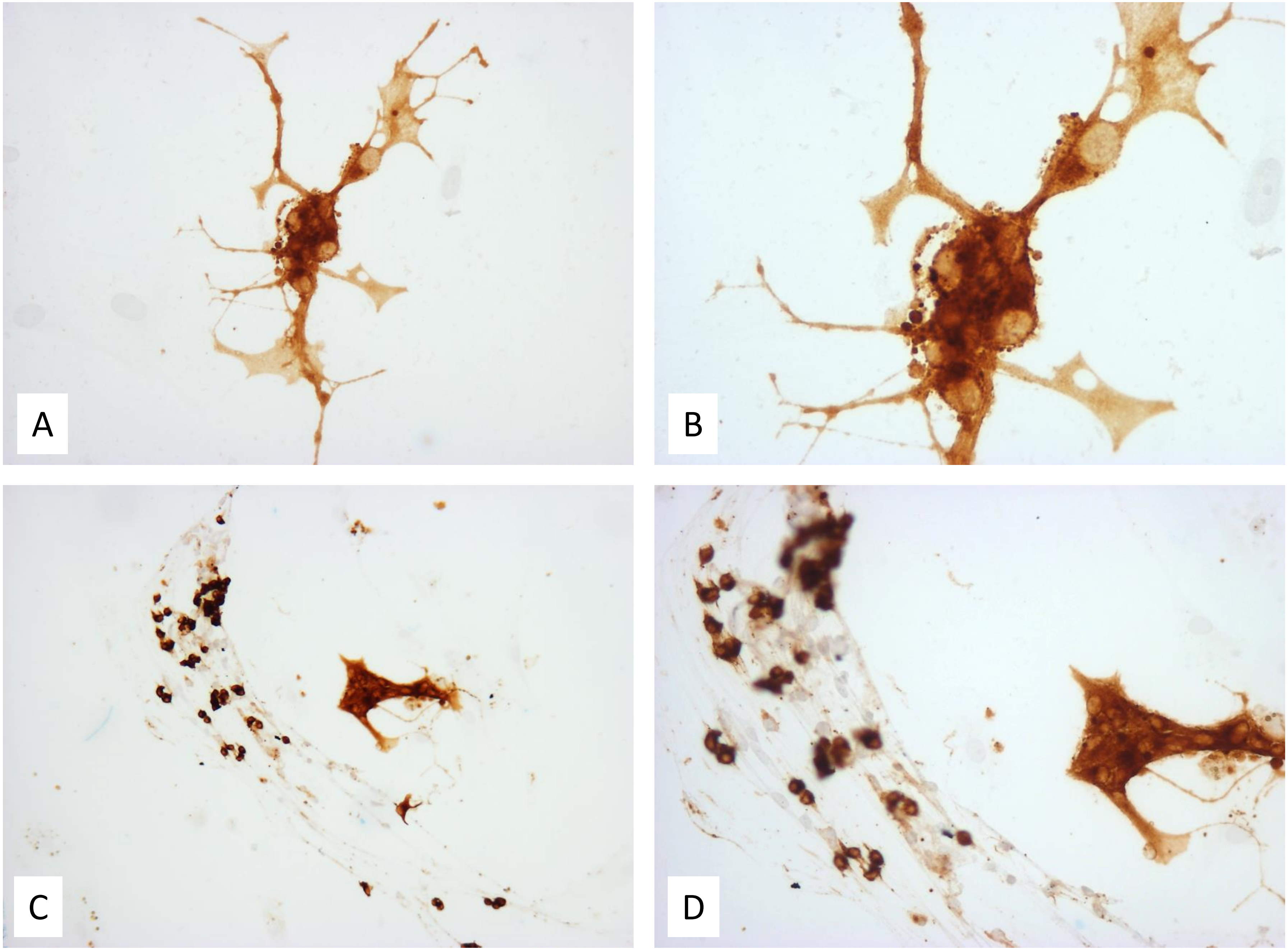
A
B
C
D

S3.18 Fig. Persistence of differentiated chromaffin cells. Chromaffin cells with a differentiated morphology persist in most tumour cultures for relatively short periods. These cells exhibit eccentric but relatively short processes and are rarely present in groups of more than a few cells after a few weeks of culture (A, Tum42_4 weeks_5% FBS_Syn_20x obj.; B, 40x obj.). Undifferentiated and differentiating cells occur concurrently in cultures (C, Tum42_4 weeks_PC12_Syn_10x obj.; D, 20x obj.) but only undifferentiated cells persist long-term. Immunocytochemical staining with synaptophysin confirms the identity of these cells.

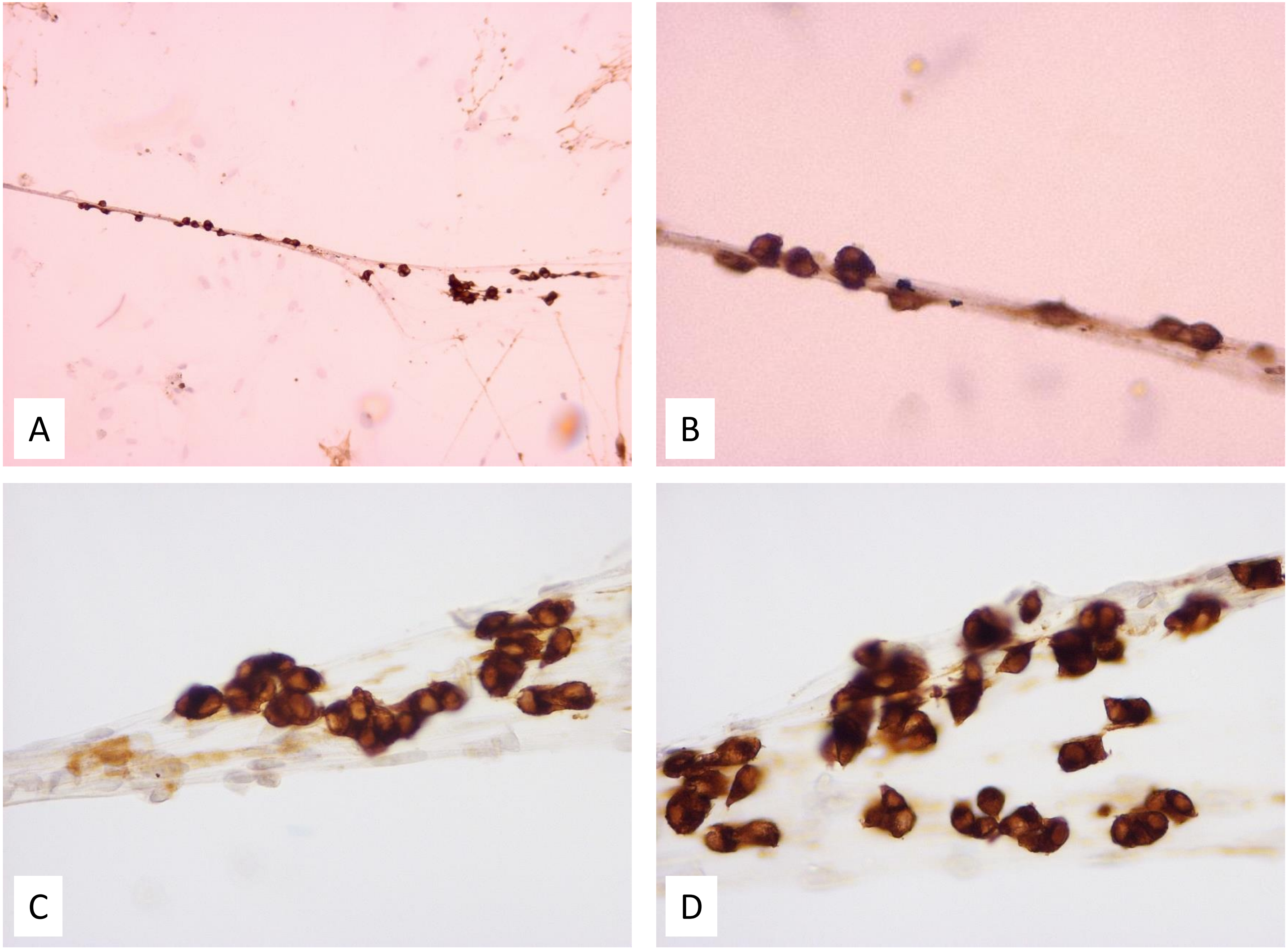
A
B
C
D

S3.19 Fig. Undifferentiated chromaffin cells. Chromaffin cells of relatively undifferentiated morphology often associate with prominent structural components in tumour cultures. Perhaps chromaffin cells avoid being subsumed under a layer of fibroblasts by attaching to these structures (which probably consist of fibroblasts/myofibroblasts) and/or benefit from a better supply of oxygen and nutrients. Alternatively, direct attachment to plastic may induce differentiation, a finite life-span and loss from culture. This behaviour is present in both relatively recent cultures (A, Tu42_4 weeks_1% FBS_Syn_10x obj.; B, Tu42_4 weeks_ 1% FBS_ Syn_ 40x obj.) and in cultures of 16 months or more (C & D, Tu18_16 months_5% FBS_Syn_40x obj.). Immunocytochemical staining with synaptophysin.

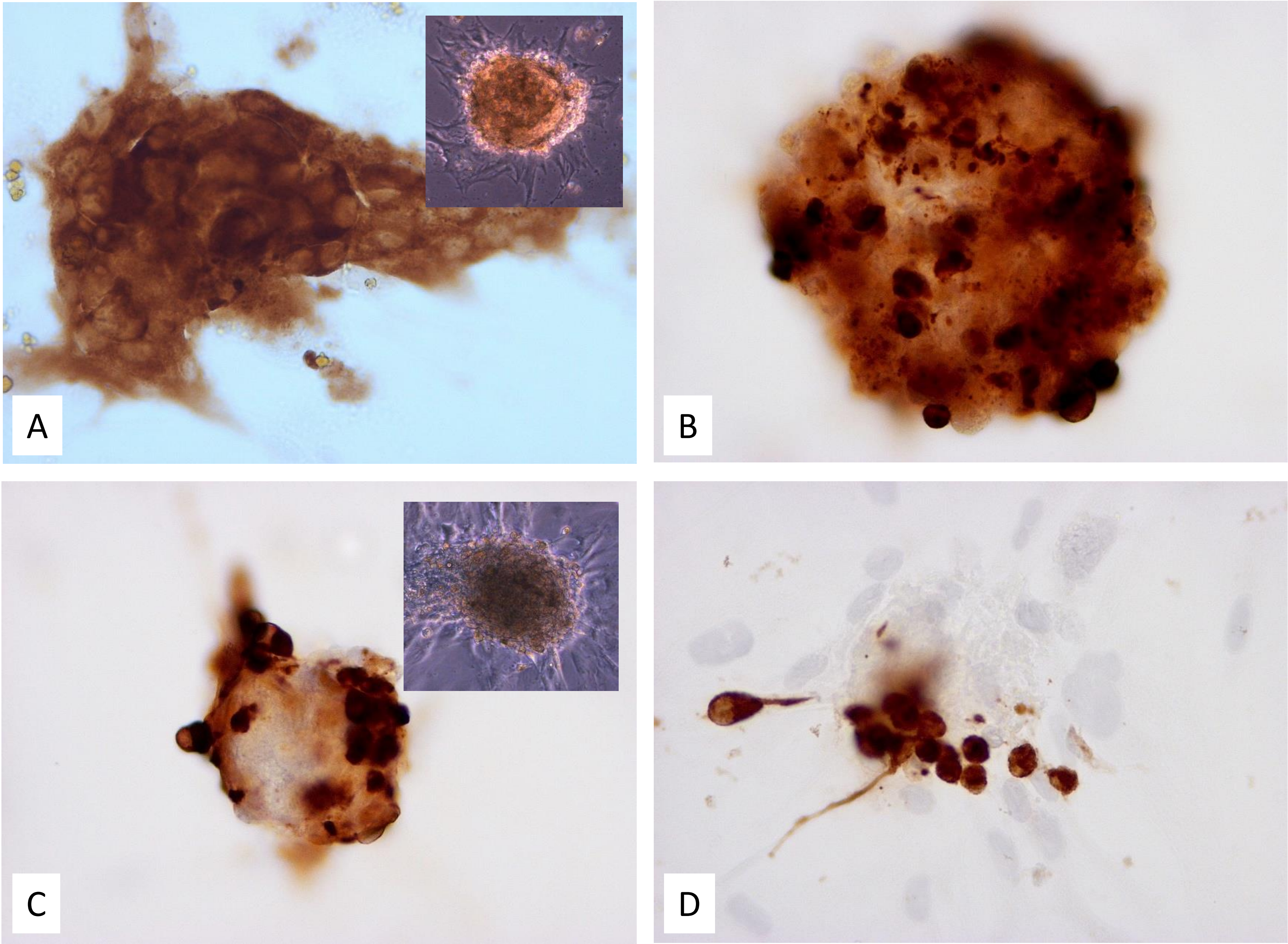
A
B
C
D

S3.20 Fig. PPGL tumour cultures often develop prominent structure. These include very large cell masses and micro and macrofibres that consist of hundreds or thousands of cells that may extend for several centimetres. Prominent cell masses (A, Tu42_2 weeks_5% FBS_TH_40x obj.; B, Tu44_4 weeks_5% FBS_Syn_40x obj.) often initially express synaptophysin but gradually loose expression and by 4-6 weeks show synaptophysin-expressing cells only in the outermost layer. Presumably the chromaffin cells inside these structures are replaced by fibroblasts or other non-synaptophysin expressing cells, although it is possibly that differentiating chromaffin cells in culture lose expression of synaptophysin over time. Some cell masses are only weakly positive (C, Tu44_4 weeks_5% FBS_Syn_40x obj.) or entirely negative (D, Tu44_4 weeks_5% FBS_Syn_ 40x obj.) for synaptophysin but have associated Syn+ cells in their outer layer. Images A and C include insets with phase contrast images of cell masses in culture. Immunocytochemical staining with synaptophysin.

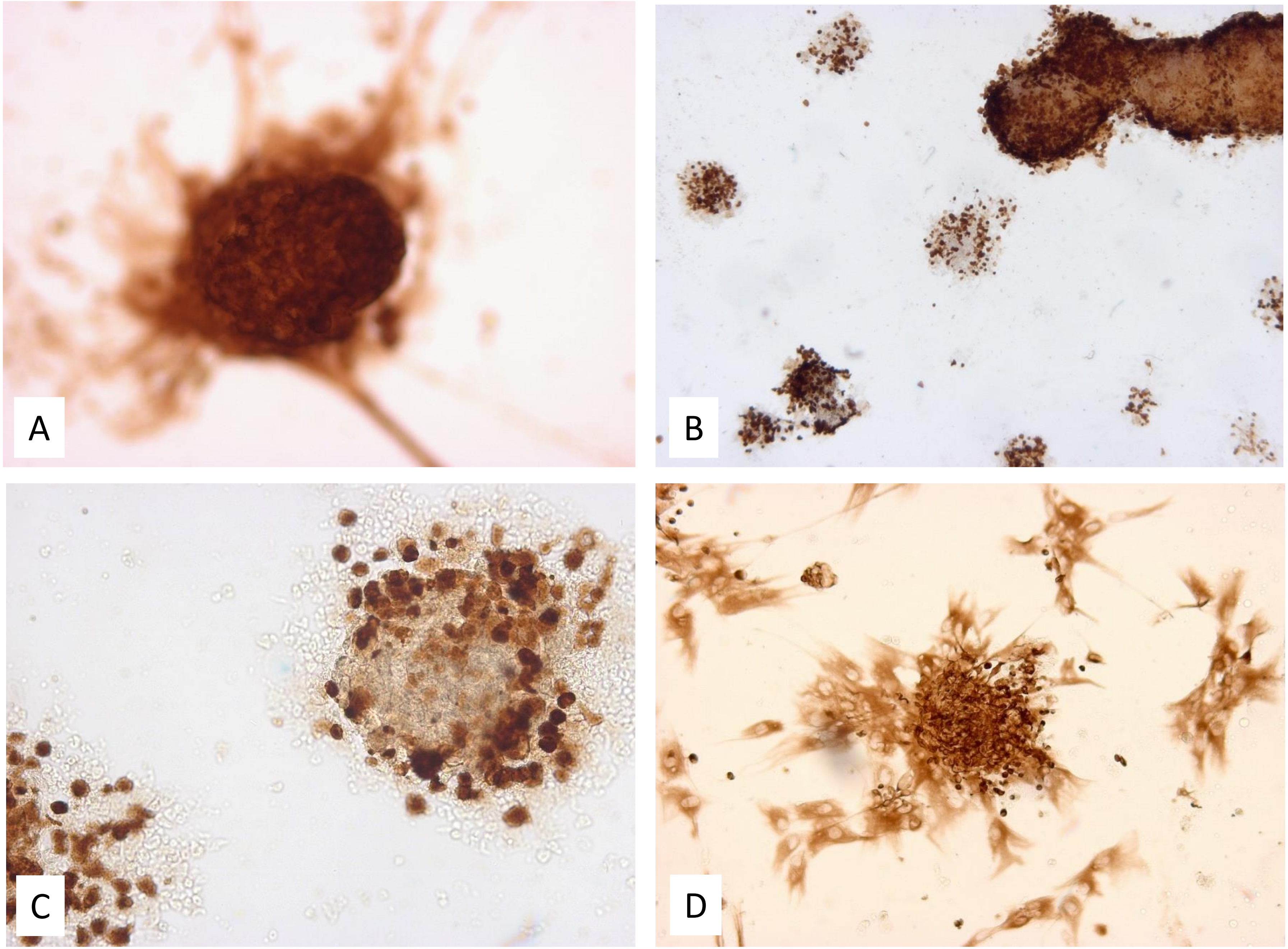
A
B
C
D

S3.21 Fig. PPGL tumour cultures often develop prominent structure. Prominent structures in paraganglioma tumour cultures often include large cell masses, consisting of numerous synaptophysin-positive cells in the first weeks of culture (A, Tu42_14 days_5% FBS_Syn_20x obj.; B, Tu44_14 days_1% FBS_Syn_10x obj.; C, Tu44_14 days_1% FBS_Syn_20x obj.). A vimentin antibody generally stains all cells in paraganglioma culture, including Syn+ cell masses (D; Tu44_14 days_1% FBS_Vim_20x obj.).

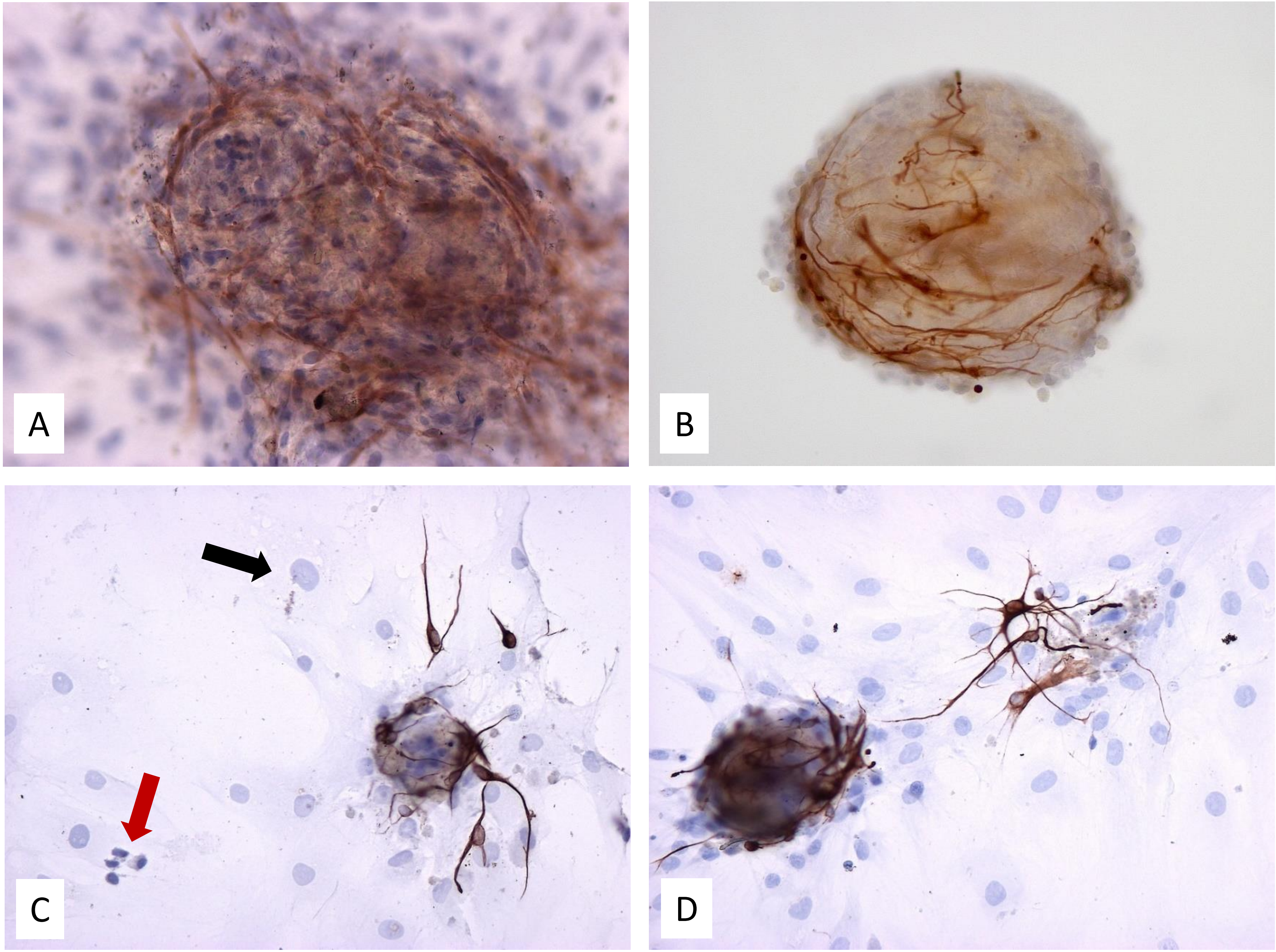
A
B
C
D

S3.22 Fig. Cells of sustentacular morphology persist in most tumor cultures for only short periods. These cells are generally characterized by eccentric but relatively short processes and often associate with (or derive from) prominent cell masses in culture (A, Tu-40_4 weeks_5% FBS_S100_20x obj.; B, Tu44_4 weeks_5% FBS_GFAP_ 20x obj.) or with smaller cell masses (C & D, Tu-40_4 weeks_1% FBS_GFAP_20x obj.). The nuclei of these cells in culture appear to be intermediate between large fibroblasts (image C, black arrow) and smaller chromaffin cells (image C, red arrow). Immunocytochemical staining with S100 (A) or GFAP (B-D) confirms the identity of these cells. Staining of older cultures (>8 weeks) with these markers tends to be entirely negative.

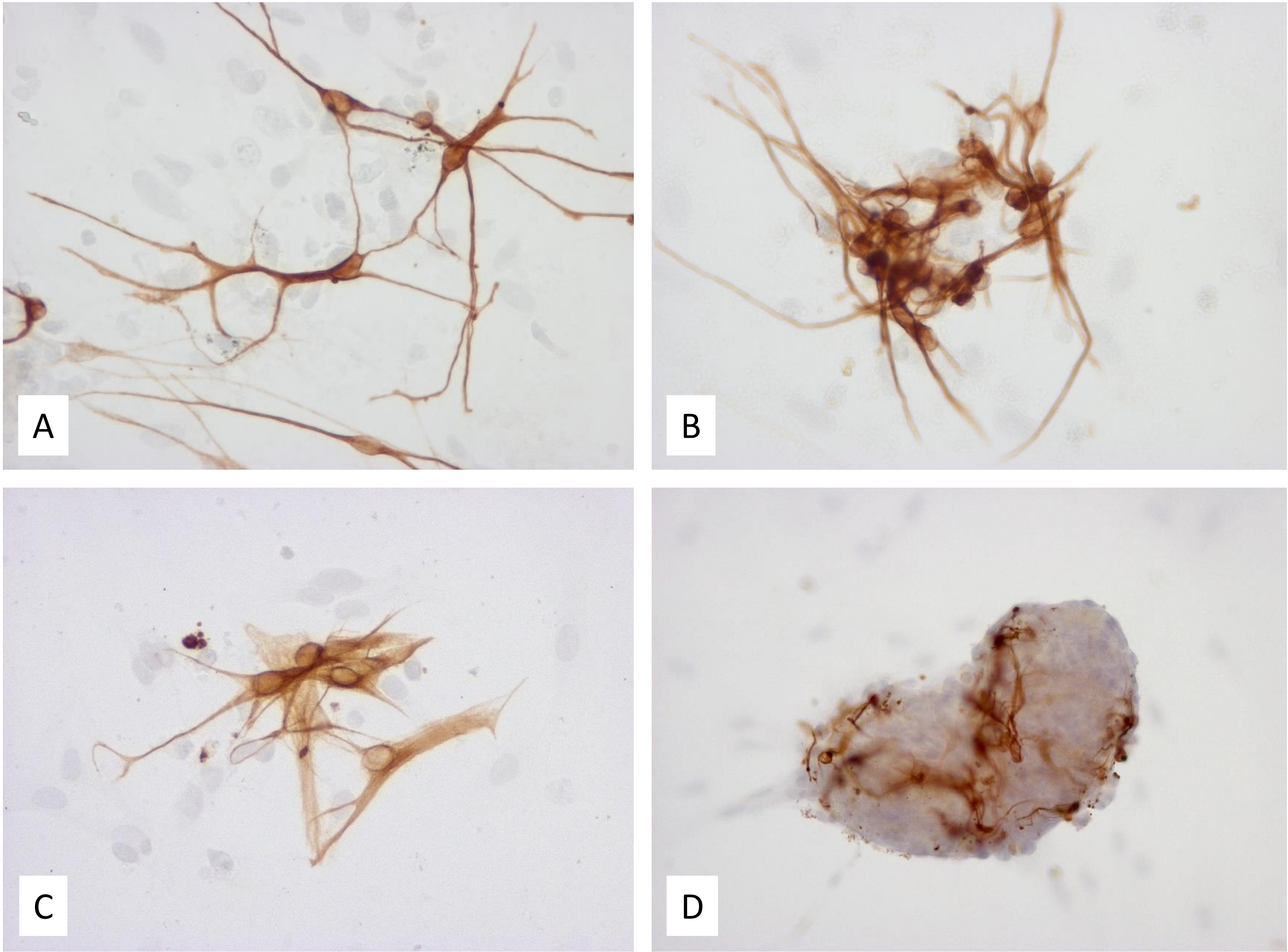
A
B
C
D

S3.23 Fig. Cells of sustentacular morphology. These cells persist in most tumour cultures for only short periods (A & B, Tu40_4 days_5% FBS_GFAP_40x obj.; C, Tu43_7 days 5% FBS_GFAP_40x obj.). Immunocytochemical staining with GFAP confirms the identity of these cells. These cells are characterized by eccentric but relatively short processes and are rarely present with more than a few cells. Staining of cultures >4 weeks with GFAP tends to reveal only sparse positivity (D, Tu44_4 weeks_5% FBS_GFAP_20x obj.) or to be entirely negative, suggesting that these cells either disappear from culture or lose expression of GFAP upon extended culture.
